# Contributions of a Child’s Built, Natural, and Social Environments to Their General Cognitive Ability: A Systematic Scoping Review

**DOI:** 10.1371/journal.pone.0147741

**Published:** 2016-02-03

**Authors:** Jazmin Del Carmen Ruiz, James J. Quackenboss, Nicolle S. Tulve

**Affiliations:** 1 Oak Ridge Institute for Science and Education, Research Participation Program, Oak Ridge, TN, United States of America; 2 Human Exposure and Atmospheric Sciences Division, National Exposure Research Laboratory, Office of Research and Development, U.S. Environmental Protection Agency, Research Triangle Park, Durham, NC, United States of America; 3 Human Exposure and Atmospheric Sciences Division, National Exposure Research Laboratory, Office of Research and Development, U.S. Environmental Protection Agency, Las Vegas, NV, United States of America; Institute for Health & the Environment, UNITED STATES

## Abstract

The etiology of a child’s cognitive ability is complex, with research suggesting that it is not attributed to a single determinant or even a defined period of exposure. Rather, cognitive development is the product of cumulative interactions with the environment, both negative and positive, over the life course. The aim of this systematic scoping review was to collate evidence associated with children’s cognitive health, including inherent factors as well as chemical and non-chemical stressors from the built, natural, and social environments. Three databases were used to identify recent epidemiological studies (2003–2013) that examined exposure factors associated with general cognitive ability in children. Over 100 factors were evaluated from 258 eligible studies. We found that recent literature mainly assessed the hypothesized negative effects of either inherent factors or chemical exposures present in the physical environment. Prenatal growth, sleep health, lead and water pollutants showed consistent negative effects. Of the few studies that examined social stressors, results consistently showed cognitive development to be influenced by both positive and negative social interactions at home, in school or the community. Among behavioral factors related to diet and lifestyle choices of the mother, breastfeeding was the most studied, showing consistent positive associations with cognitive ability. There were mostly inconsistent results for both chemical and non-chemical stressors. The majority of studies utilized traditional exposure assessments, evaluating chemical and non-chemical stressors separately. Collective evidence from a limited number of studies revealed that cumulative exposure assessment that incorporates multiple chemical and non-chemical stressors over the life course may unravel the variability in effect on cognitive development and help explain the inconsistencies across studies. Future research examining the interactions of multiple stressors within a child’s total environment, depicting a more real-world exposure, will aid in understanding the cumulative effects associated with a child’s ability to learn.

## Introduction

Despite progress over the past decades in improving children’s cognitive outcomes in the U.S. with lead poison prevention [1] and early childhood development programs [2], disparities in cognitive development in the U.S. persist. Successive waves of studies repeatedly show children from low-income families and minority races/ethnic groups continue to be at greatest risk [3–6]. This growing disparity has driven children’s research to shift towards a transdisciplinary approach [7] to considering the impact of cumulative toxic stress from chemical and non-chemical (e.g., economic, prenatal stress, parent child interaction) stressors within the total environment (i.e., built, natural and social environments).

Cognitive development during childhood is a ladder of successive stages with a network of interactions within and between stages that culminates from the continuous experiences with the environment [8]. There is, however, a critical period of time (i.e., 3–6 years old) when a child is primed to develop specific cognitive skills essential for later learning provided the right environmental variables are present [9, 10]. While peak influence on developing skills is during these defined critical periods, research in animal models does suggest that the plasticity of the brain may allow for influence beyond the critical period allowing for later intervention [11]; holding promise for similar changes in children.

Because of its dynamic nature, there is no single factor or single window of exposure which determines a child’s potential cognitive ability; rather, it is a result of real-world exposure over time to a mixture of factors with cumulative or interactive effects beginning with maternal exposures prior to conception. The U.S. Environmental Protection Agency (EPA) recognizes that children are more vulnerable to both chemical and non-chemical stressors, positive and negative, in their homes, schools and where they play, as compared to adults. As part of its commitment to protecting children’s health and well-being, EPA’s strategy includes equipping communities with tools and models that forecast the impact of real-world exposures to chemical and non-chemical stressors in the total environment.

The purpose of this manuscript is to provide a systematic scoping review of positive and negative stressors associated with children’s general cognitive ability that are linked to a child’s total environment; thus serving as a source of evidence for tailoring existing tools or models used by communities that consider cognitive development a critical area of concern. Previous reviews of child development have considered exposures within their respective discipline (i.e., social or physical; chemical or non-chemical) [12–17], with the exception of one review focused on children from developing countries [18]. This review aims to identify both protective and risk factors of general cognitive ability using a more encompassing model of environmental exposure and review the state-of-the-science in addressing the complex and interactive effects of these factors.

## Methods

### Data Source

To identify studies eligible for inclusion in the review, a search of the peer-reviewed literature was conducted using the following databases: PubMed, Web of Science, PsycINFO. A combination of words associated with cognitive health (e.g., neurodevelopment or cognitive development) OR ((cognition or learning) AND association) AND children were used to form search strings and applied in the literature search. Filters were used to limit the search to studies published within the past 10 years (2003–2013), available in English, and conducted in children from birth to 18 years of age.

### Study Selection

The publications found using the search strings and limitations went through three rounds of screening. In the first round, titles were screened and selected based on their relevance to the research question. In the second round, abstracts of the selected titles were reviewed based on whether they met the inclusion criteria. Finally, full text articles of the selected abstracts were retrieved for further assessment of relevance to the research question. The top ten PubMed related citations of the articles that met the inclusion criteria were also reviewed.

#### Inclusion Criteria

The following criteria were used to determine whether a study was eligible to be included in this review.

- Observational studies, randomized control trials, review or meta-analysis;
- Time of exposure to a determinant or stressor occurred at or before health outcome was assessed;
- Health outcome was measured in children under 18 years old;
- General cognitive outcome was measured using current and earlier versions of evidence-based assessments of cognitive functioning classified as well established [19] and expressed as a continuous variable or categorized as below average or significant cognitive delay (i.e., >1 or >2 standard deviations (SD) below the mean).
- Study included a measure of association and statistical significance;
- Majority of study participants were healthy children without any existing developmental disabilities, neonatal morbidities, pathologies associated with cognitive deficits, or rare disorders.

### Data Extraction and Synthesis

Information extracted from the eligible studies included: author(s) and year of publication, name of study (if available) or the country in which the study was conducted, number of children evaluated, exposure factors and cognitive health outcome measured, the average age(s) at which these measures were assessed, covariates used in the analysis and study results (i.e., means and SD, regression or (B or β) Pearson correlation (r) coefficients, odds ratios (OR)) and indicators of statistical significance (i.e., 95% CI, p-values). Study results include those from univariate (univ) and multivariate analyses for the exposure of interest (EOI) and all possible independent predictors (IP). Factors were categorized into three broad domains: 1) individual determinants; 2) social environment, and 3) built and natural environments A narrative review is provided for each factor assessed in two or more studies either as the EOI or an IP included in the multivariate analysis with consistent results. Results were reported as point differences or B when comparing studies with similar cognitive score SD (i.e., mean = 100, SD = 15–16) and similar exposure measures; otherwise, differences in SD of the cognitive score, β or r are used.

## Results

**Fig 1:**
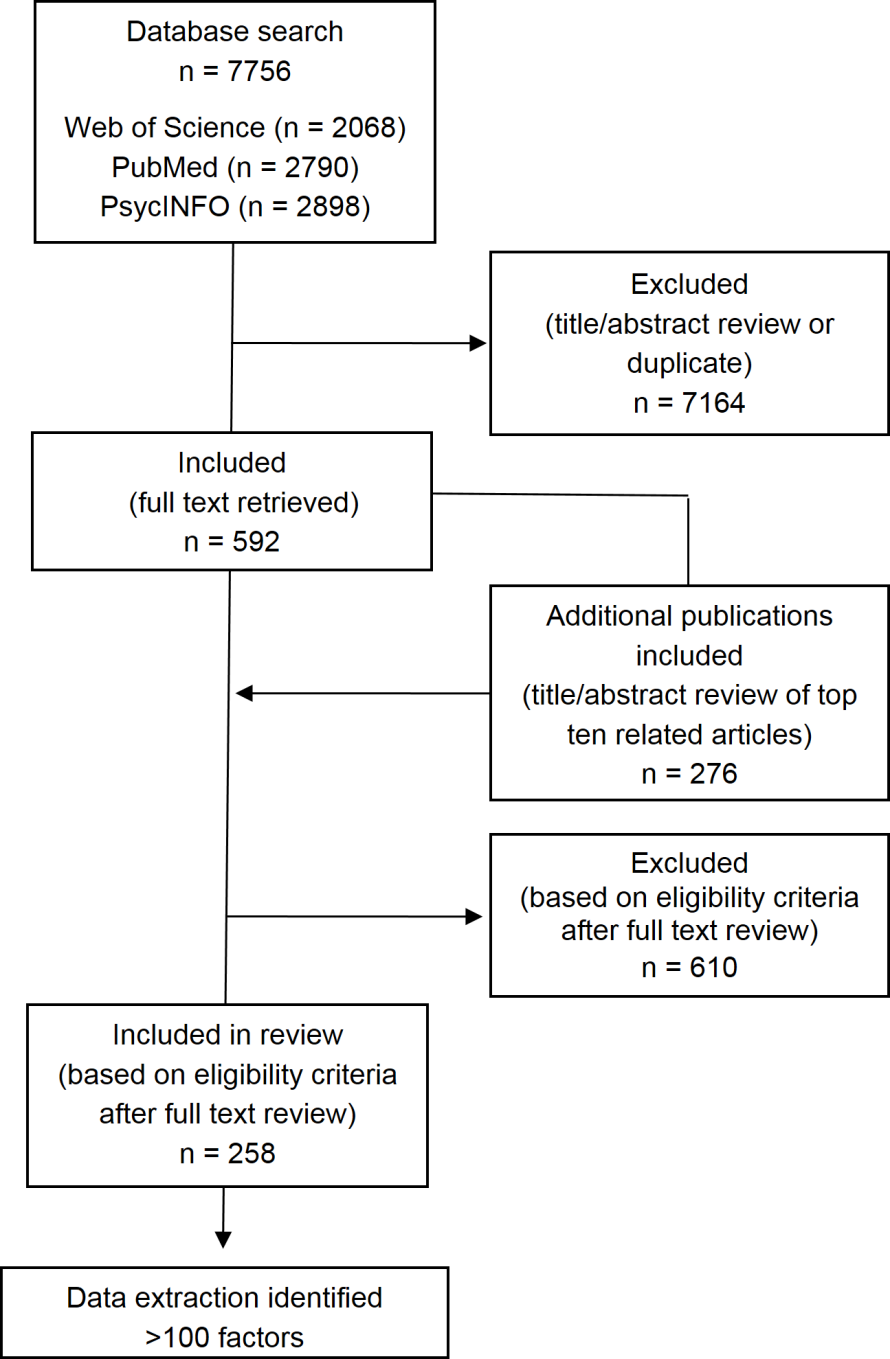
Flowchart of selection process for review.

A total of 133 observational studies and 13 cohorts nested within randomized controlled trial studies conducted in 38 countries met the inclusion criteria. Four studies included in this review were pooled data or meta-analysis studies, two of which included cohorts from nine of the observational studies. The most common reasons for exclusion was not reporting results for general cognitive outcome or not using one of the specified tools to measure the outcome. A summary of characteristics of these studies are presented in Table 1. S1 Table provides a summary of the 258 publications [20–281], grouped by study, included in this review. Over 100 unique factors were examined across the studies. A flowchart of the selection process is illustrated in Fig 1. Table 2 lists a summary of results for all determinants/stressors presented in two or more studies. S2 Table lists the factors that were examined in only one study.

**Table 1:** Summary of Characteristics of the Included Studies.

Type of Study		*n*
Pooled Analysis		3
Meta-analysis		1
Cohort Studies		
	Prospective	105
	Retrospective	3
	Cross-Sectional	38
Average Sample Size		
	<100	27
	100-<1000	103
	1000-<10000	14
	≥10000	2
First Year of Recruitment		
	<1990	19
	1990–1999	53
	≥2000	41
	Not Reported	33
Location of Study		
	U.S, Canada	61
	Latin America	8
	Europe	40
	Australia	13
	Asia	19
	Africa	2
	International	3

**Table 2:** Factors Examined in Multiple Studies as EOI or IP of General Cognitive Ability.

Factor		Number of Studies	Total *n*	Results Across Studies			Overall Trend of Association (% of studies)
				Positive	Negative	Null	
**Demographics**							** **
Age (Maternal) [22,23,27,41,43,49,56–60,75,78,82–84, 86,100,127]		19	10187	4	1	14	No trend (74)
Ethnicity/Race							
	All minorities vs white [20,21]	2	8138	0	1	1	Inconsistent
	Non-Hispanic black vs non-Hispanic white or non-black [22–25,27–30]	8	5694	0	8	0	Negative (100)
	Hispanic vs non-Hispanic white [23,24,27]	3	3393	0	1	2	No trend (67)
	Other Minorities (Asian, Native American, mixed, other) vs non-Hispanic white [22,24,26]	3	1504	0	0	3	No trend (100)
	Non-Hispanic black versus Hispanic [32,35]	2	293	2	0	0	Positive (100)
Education							
	Maternal [23,27,29,37–49,56–66]	27	10716	17*	1	9	Positive (63)
	Paternal [41,58,61,63,67]	5	1622	3	0	2	Positive (60)
Employed [27,30,46,61,75]		5	3548	1	0	4	No trend (80)
Income							
	Household [20,23,27,41,46,57,58,66,249]	9	12379	4	0	5	Inconsistent
	Low income [22,27,36,272]	4	4462	1	1	2	Inconsistent
Resources							
	Household assets [65,70,71]	3	2734	3*	0	0	Positive (100)
	Lack of resources, material hardship [22,31,42,84,126]	5	1550	2*	0	3	Inconsistent
Additional Socioeconomic Status (SES) Factors							
	Occupational class [20,48,63,72,74,75]	6	10676	5*	1	0	Positive (83)
	Hollingshead four factor index [80,82,84,94,128,170]	6	1202	3*	0	3	Inconsistent
	Other SES index/composite [22,24,26,47,61,85,88,157,224]	9	6509	5	0	4	Positive (56)
Language (English is dominant language) [22,23,30,35]		4	3226	0	0	4	No trend (100)
Parent Origin (Immigrant) [58,75]		2	2288	0	0	2	No trend (100)
**Inherent Factors-Child**							
Gender (female vs male) [22,23,27,29,30,37,38,40,41,43,48, 54,56,58,62,63,66,70,71,75–77,80–89]		32	17465	20	2	10	Positive (63)
Anthropometry							
	Birthweight (BW) [20,24,41–43,48,58,73,79,82,84,85, 102,141,171]	15	23395	7*	1	7	Inconsistent
	Low BW [23,70,71,75,90, 91, 93–97]	11	5871	0	10	1	Negative (91)
	Current body mass index (BMI) [60,80,101] or weight [98]	4	884	0	2*	2	Inconsistent
	Below average BMI or weight [66,219]	2	811	0	1	1	Inconsistent
	Current height [39,47,64,65,98,101]	6	1324	5	0	1	Positive (83)
	Current head circumference [64,65,100,101]	4	1262	4	0	0	Positive (100)
	Growth over time [49,102,103]	3	12922	3	0	0	Positive (100)
	Poor growth over time [55,251]	2	5935	0	1	1	Inconsistent
Birth Outcomes and Neonatal Health							
	Gestation (weeks) [20,31,40,48,75,83,127,128,170]	9	10415	3	1	5	No trend (56)
	Preterm (<37 weeks) [58,60,104,105]	4	1021	0	2	2	Inconsistent
	Moderate to late preterm (32-<37 weeks) [106–108]	3	12785	0	1	2	No trend (67)
	Extremely preterm (<32 weeks) [109–111]	3	3020	0	3	0	Negative (100)
	Neonatal ICU [58,267]	2	903	0	1	1	Inconsistent
	Neonatal medical risk [86,97]	2	354	0	1	1	Inconsistent
	Resuscitation [221,263]	2	6075	0	1	1	Inconsistent
Sleep Behavior and Health							
	Sleep disordered breathing (SDB) [112–119]	8	2180	0	7*	1	Negative (88)
	SDB symptom (snoring) [114,116,117,120]	4	356	0	4	0	Negative (100)
	Daytime sleepiness [121,122]	2	674	0	2*	0	Negative (100)
	Sleep wake problems [80,122]	2	205	0	0	2	No trend (100)
	Duration [80,118,122,234]	4	1100	2	1	1	Positive (50)
Childhood Health							
	Health history [28,84,88]	3	5509	0	1	2	No trend (67)
	Atopic disease [74,252]	2	691	0	1	1	Inconsistent
	Eczema [243,252]	2	614	0	1	1	Inconsistent
	Thyroid biomarkers (TSH level) [217,229,241,258]	4	1363	0	2	2	Inconsistent
**Inherent Factors-Parents**							
Maternal IQ [20,23,24,27–30,37,60,61,64,65,72,79,81–87,89,100,123–126,128]		28	18768	24	0	4	Positive (86)
Physical Health (prenatal)							
	Multiple gestation [20,58,83,97]	4	8477	0	3	1	Negative (75)
	Parity [20,22,26,54,57,62,63,74,78,83,124, 127]	12	11640	0	5	7	No trend (58)
	Body Mass Index or weight (pre-pregnancy) [20,60,75,133]	4	16418	0	4	0	Negative (100)
	Thyroid dysfunction (low T4) [129–132]	4	2224	0	3	1	Negative (75)
	Thyroid dysfunction (TPOAb) [83,129]	2	183	0	1	1	Inconsistent
	Thyroid dysfunction (high TSH) [83,129,130,258]	4	2431	0	1	3	No trend (75)
	Thyroid biomarkers (TSH level) [222,241,258]	3	1065	0	0	3	No trend (100)
	Thyroid biomarkers (T4 level) [222,241,258]	3	1065	0	0	3	No trend (100)
	Dental amalgam [82,277]	2	828	1	0	1	Inconsistent
	Hypertension [128,278]	2	1497	0	0	2	No trend (100)
Maternal Mental Health							
	Anti-depressants (prenatal) [125,232]	2	281	0	0	2	No trend (100)
	Prenatal depression (high risk) [125,134,135]	3	7198	0	1	2	No trend (67)
	Prenatal depression (symptom score) [125,135]	2	463	0	0	2	No trend (100)
	Postpartum depression(high risk) [134–136]	3	7565	0	2*	1	Negative (67)
	Postpartum depression (symptom score) [27,57,135]	3	1900	0	2*	1	Negative (67)
	Postnatal depression (high risk) [46,83,134]	3	5215	0	1	2	No trend (67)
	Postnatal depression(symptom score) [27,28,57,125]	4	2318	0	1	3	No trend (75)
	Prenatal stress [43,137,138]	3	825	0	3*	0	Negative (100)
	Postnatal stress [43,83,138]	3	877	0	0	3	No trend (100)
	Prenatal anxiety [43,135,139]	3	473	0	1	2	No trend (67)
	Prenatal cortisol [43,137,139]	3	335	0	3	0	Negative (100)
**Diet**							
Breastfeeding							
	Ever breastfed [56,141,154]	3	1623	3	0	0	Positive (100)
	Breastfeeding duration [20,48,52,60,61,63,74,83,142,146, 147]	11	10191	8*	0	3	Positive (73)
	Exclusively breastfed [54,148,150]	3	2375	3*	0	0	Positive (100)
	Breastfeeding intensity [148,149]	2	7601	2*	0	0	Positive (100)
	Total n-3 long chain-polyunsaturated fatty acid (LC-PUFA) [82,148]	2	745	1	0	1	Inconsistent
	Docosahexaenoic acid (DHA) [48,148,151–153]	5	1018	3	0	2	Positive (60)
	Total n-6 LC-PUFA [82,148]	2	745	0	0	2	No trend (100)
	Arachidonic acid (AA) [48,148,151,153]	4	938	1	1	2	Inconsistent
	Linolenic acid [48,148]	2	577	0	0	2	No trend (100)
	DHA/AA ratio [48,148,152]	3	653	2	0	1	Positive (67)
	α-linolenic acid [48,148]	2	577	0	0	2	No trend (100)
	Eicosapentaenoic acid [48,148]	2	577	0	0	2	No trend (100)
Child’s Diet							
	Infant diet pattern, recommended/healthy [149,155]	2	7338	2	0	0	Positive (100)
	Infant diet pattern, not recommended, processed [149,155]	2	7338	0	1	1	Inconsistent
	All fish [71,144]	2	226	0	1	1	Inconsistent
	Canned fish [71,144]	2	226	1	0	1	Inconsistent
	Dietary iron [38,40,47,87,100,157]	6	1292	4	0	2	Positive (67)
Prenatal Diet							
	Any seafood [237,247,253,258]	4	10082	2	0	2	Inconsistent
	Fish [71,75,233,253]	4	2431	2	0	2	Inconsistent
	Choline [29,281]	2	486	1	0	1	Inconsistent
	Folate supplementation [159,160]	2	973	0	0	2	No trend (100)
	Folate deficiency [127,158]	2	508	1	0	1	Inconsistent
	Iodine supplementation or intake [258,265]	2	2201	0	0	2	No trend (100)
**Lifestyle Factors**							
	Alcohol (pre-conception) [161,239]	2	412	0	1	1	Inconsistent
	Alcohol (prenatal, any exposure) [20,25,42,43,78,84,86]	7	9438	0	1	6	No trend (86)
	Alcohol (prenatal, frequency) [161,164,216, 240,280]	6	19516	0	2	4	No trend (67)
	Low alcohol intake (prenatal) [162,163,165]	3	9020	0	0	0	No trend (67)
	Moderate to high alcohol intake (prenatal) [161–163,165]	4	9095	1	3	0	Negative (75)
	Binge drinking (prenatal) [164,165,216,262]	4	18567	0	1	3	No trend (75)
	Alcohol (father, frequency) [169,216]	2	24549	1*	1	0	Inconsistent
	Alcohol related dependence (father) [28,166]	2	680	0	2	0	Negative (100)
	Maternal smoking (prenatal, any) [41,56,60,63,71,78,83,242]	8	2526	0	1	7	No trend (88)
	Maternal smoking (prenatal, frequency) [42,43,86,216, 226,242]	6	16345	0	1	5	No trend (83)
	Maternal smoking (postnatal, any) [61,242]	2	584	0	0	2	No trend (100)
	Maternal smoking (postnatal, frequency) [42,242]	2	590	0	0	2	No trend (100)
	Environmental tobacco smoke (ETS) (prenatal, any) [31,50,62,126,242]	5	1372	0	2	3	No trend (60)
	ETS (prenatal, frequency) [44,216,242]	3	14314	0	1	2	No trend (67)
	ETS (postnatal, any) [41,52,62]	3	972	0	0	3	No trend (100)
	Cocaine (prenatal) [25,42,77,86,124,248,254,257,268]	9	3322	0	3	5	No trend (63)
	Marijuana (prenatal) [28,42,86]	3	1049	1	0	2	No trend (67)
**Social Environment**							
Family Structure							
	Marital status [20,27,30,31,124,126,127,169]	8	22484	4*	0	4	Inconsistent
	Live with both parents [23,82]	2	2330	1	0	1	Inconsistent
	Primary caregiver is mother [42,75]	2	1853	1	0	1	Inconsistent
	Foster or institutional care [167,168]	2	420	0	2	0	Negative (100)
	Siblings [20,25,27,58,61]	5	9595	0	4*	1	Negative (80)
	Changes in family structure [22,169]	2	11956	0	2	0	Negative (100)
Parent Interactions							
	Cognitive stimulation–activities [26,88]	2	4920	2	0	0	Positive (100)
	Cognitive stimulation-HOME [20,27,37,59,64,70,72,76,81,84,87,89,124,141,170]	15	12490	15****	0	0	Positive (100)
	Cognitive stimulation–HSQ [28,29,63,136]	4	1632	3	1	0	Positive (75)
	Cognitive stimulation–PROCESS [25,82]	2	500	2	0	0	Positive (100)
	Cognitive stimulation—other tool [22,84,86]	3	1287	2	0	1	Positive (67)
	Quality of maternal interaction [23,35,88,170]	4	6366	4	0	0	Positive (100)
	Sensitivity [22,27,67,69,97,171]	6	1611	3	0	3	Inconsistent
	Supportive behavior [26,88,170,171]	4	5036	3	0	1	Positive (75)
	Discipline [22,88,170]	3	4557	0	0	3	No trend (100)
Social support for the mother [28,138]		2	1171	2	0	0	Positive (100)
Childcare/School							
	School attendance [64,100]	2	595	2	0	0	Positive (100)
	Child care attendance [27,46]	2	974	0	0	2	No trend (100)
	Type of child care (center or group) [22,27]	2	1260	2	0	0	Positive (100)
	Quality of care [22,27,69]	3	1323	3	0	0	Positive (100)
Additional Psychosocial Factors							
	Witnessed domestic violence [66,245]	2	2384	0	1	1	Inconsistent
	Neglect or abuse [68,224]	2	662	0	0	2	No trend (100)
	Stress/anxiety [48,68,83,224]	4	832	0	1	3	No trend (75)
**Built and Natural Environments**							
Metals and Other Elements							
	Arsenic (As) (current) [47,64,65,100,173,175,176]	7	3725	1*	5	1	Negative (71)
	As (prenatal) [175,176]	2	2149	0	1*	1	Inconsistent
	Cadmium [186,190]	2	1297	0	0	2	No trend (100)
	Fluoride (current) [47,174,178–180]	5	1475	0	5	0	Negative (100)
	Lead (Pb) (current, sample restricted to ≤10 μg/dL) [24,41,79,157,184,185]	6	1588	0	5	1	Negative (83)
	Pb (current, no restriction) [38,47,85,87,182,183, 185,187–189]	10	2316	0	8*	2	Negative (80)
	Pb (prenatal) [31,52,59,85,98,127,190]	7	2111	0	5	2	Negative (71)
	Pb (lifetime) [59,85,183,187]	4	1120	0	3	1	Negative (75)
	Pb (postnatal) [85,183,187,188]	4	1481	0	1	3	No trend (75)
	Manganese (postnatal) [38–41,64,99,100,188,192]	8	1906	1	6*	1	Negative (75)
	Mercury (Hg) (prenatal) [51,71,75,218,223,247,271]	7	4436	0	4*	3	Positive (57)
	Hg (postnatal) [30,71,84,144]	4	1664	1	1	2	Inconsistent
Air Pollutants							
	Nitrogen dioxide (NO2) (indoor) [74,193]	2	2305	0	2	0	Negative (100)
	NO2 (outdoor) [230,236]	2	2099	0	1	1	Inconsistent
	Polyaromatic hydrocarbons (prenatal) [37,50,126]	3	561	0	3	0	Negative (100)
Endocrine Disruptors							
	Total polybrominated diphenyl ether (PBDE) (prenatal) [194,195]	2	267	0	2*	0	Negative (100)
	Total PBDE (postnatal) [194,196,197]	3	608	0	1	2	No trend (67)
	PBDE 47 (prenatal) [195,198,199]	3	254	0	1	2	No trend (67)
	PBDE 47 (postnatal) [196,198,199]	3	600	0	0	3	No trend (100)
	PBDE 99 (prenatal) [95,198]	2	176	0	2	0	Negative (100)
	PBDE 99 (postnatal) [196,197]	2	360	0	1*	1	Inconsistent
	PBDE 100 (prenatal) [195,198]	2	176	0	1	1	Inconsistent
	PBDE 100 (postnatal) [196,197]	2	360	0	0	2	No trend (100)
	PBDE 153 (prenatal) [195,198]	2	133	0	1	1	Inconsistent
	PBDE 153 (postnatal) [196,197]	2	360	0	0	2	No trend (100)
	PBDE 154, 183 (postnatal) [196,197]	2	360	0	0	2	No trend (100)
	PBDE 209 (postnatal) [196,197]	2	360	0	2	0	Negative (100)
	Phthalate [200–202]	3	1145	0	2	1	Negative (67)
	Total PCBs (polychlorinated biphenyl) (prenatal) [89,212,227,250,260,271]	6	2845	0	2	4	No trend (67)
	PCB 118 (prenatal) [89,227,260,270]	4	1381	0	1	3	No trend (75)
	PCB 138 (prenatal) [89,212,227,270]	4	2638	0	1*	3	No trend (75)
	PCB 153 (prenatal) [89,212,227,250,270]	5	2670	0	2*	3	No trend (60)
	PCB 153 (postnatal) [227,250]	2	306	0	0	2	No trend (100)
	PCB 156 (prenatal) [89,260]	2	894	0	1	1	Inconsistent
	PCB 170 (prenatal) [89,260]	2	894	0	0	2	No trend (100)
	PCB 180 (prenatal) [89,212,227,260,270]	5	2772	0	1*	4	No trend (80)
	PCBs+dioxins (prenatal) [260,279]	2	284	0	0	2	No trend (100)
Pesticides							
	Mirex (prenatal) [203,204]	2	262	0	0	2	No trend (100)
	Chlorpyrifos (prenatal) [205,206]	2	635	0	1	1	Inconsistent
	Dichlorodiphenyltrichloroethane (DDT) (prenatal) [204,209–212]	5	2528	0	3	2	Positive (60)
	Hexachlorobenzene (prenatal) [204,212]	2	1549	0	1	1	Inconsistent
	Dialkylphosphates (DAP) (prenatal) [205,208,214]	3	599	0	2	1	Negative (67)
	DAP (postnatal) [205,208]	2	450	1	0	1	Inconsistent
House quality [65,174,206]		3	2481	1	1	1	Inconsistent

* indicates number of studies that were significant after stratified analysis

### Individual Determinants

Individual determinants of health and well-being include inherent biological (e.g., health, genetics) and social demographic characteristics of the child and parent. Individual determinants also include behaviors or activities of the child (e.g., physical activity, diet) and the parent (e.g., diet, smoking, drugs). However, it is important to note that levels of exposure to such behaviors and activities are factors that may be influenced by lifestyle choices and modelling by parents or other elements of the social environment. Determinants at this level may also impact the extent of exposure and vulnerability to stressors within the social, built and natural environments; making it necessary to examine the embedded heterogeneous effects of both inherent and behavioral stressors of cognitive outcome with reference to exogenous environmental contexts.

#### Household Demographics

Eleven U.S. studies and one UK study included maternal race/ethnicity in their analyses (IP n = 11). In general, minority or non-white children when lumped into one group had lower cognitive scores than white children (*P*_*univ*_*(s)*<0.05) [20, 21]. Differences in effect were found in studies that separated minorities into multiple groups. The cognitive scores of black children measured as early as one year old were two to ten points lower than white children in eight studies in which the effects of race were independent of EOIs (i.e., illicit drug or lead (Pb) exposure, choline, child care, maternal employment, parenting quality, and cognitive stimulation) (B(s) = -9.9 to -1.7, *P(s)*<0.05) [22–29]. Similar differences in cognitive scores were noted in a U.S. cohort of Pb-exposed children (>20 μg/dL) when black children were compared to non-black children (B = -3.0, *P*<0.05) [30].

In comparisons of Hispanic versus white children, the negative association was weaker after adjusting for similar exposures (*P*<0.05 [27]; *P(s)*>0.05 [23, 24, 26]). The disadvantage may be stronger in Hispanic children living in urban cities as evidenced in two U.S. studies evaluating cognitive development. Hispanic children had lower scores assessed at earlier ages as compared to non-Hispanic black children (i.e., 4–6 points) independent of EOIs (i.e., parent child interactions, pesticide exposure and air pollution) (*P(s)*<0.05) [31–35]. However, the effect diminished with age; whereas, the effects of air pollution and pesticide exposure persisted (*P(s)*>0.05) [36, 37].

Maternal education (i.e., years or level of attainment) was included in 27 studies as a possible predictor of cognitive ability (EOI n = 2; IP n = 25). Maternal education was measured in years in sixteen studies (EOI n = 1; IP n = 15). In a low-income U.S. cohort, years of education continued to be a positive predictor of early cognitive development even after accounting for other significant positive predictors (i.e., economic resources and parenting) (B = 0.11; *P*<0.05) [23]. Maternal education can have a protective influence in children exposed to manganese (Mn) as evidenced in four studies where cognitive scores increased 0.7–1.0 point per year of education (*P(s)*<0.05 [38–40]; *P* = 0.08 [41]). In fact, the benefit associated with maternal education was strongest in Korean children with high Mn levels (>14 μg/dL) (B = 1.72, *P*<0.05) [41]. In one U.S. study that used several models to evaluate the effects of choline, level of education was a consistent positive predictor of cognitive ability (B(s) = 0.9; *P(s)*<0.05) [29]. Years of education was also a positive predictor in six studies independent of their EOIs (i.e., employment, stress, cocaine, Pb, pesticide and indoor mold) ((+) *P(s)*<0.05 [27, 36, 42–45]; (-) *P*<0.05 [46]; *P(s)*>0.05 [47–49]).

Thirteen studies included level of educational attainment as a predictor of cognitive ability (EOI n = 1; IP n = 12). Three longitudinal cohort studies of children from the U.S. [31, 32, 34, 36, 37], Poland [44, 50–54] and the UK [20, 55, 56] consistently found level of education to be a positive predictor of cognitive development over time regardless of the EOI. Seven studies suggested that completion of secondary education (at a minimum) benefitted cognitive development for children from higher income countries independent of EOI (B(s) = 1.3–7.5; *P(s)*<0.05 [37, 54, 56–60]; *P(s)*>0.05 [61–63]). In children from low income countries any education above the primary level (vs. no education or illiteracy) may positively influence cognitive ability, as suggested by two studies examining the effects of water contamination on cognitive development in Bangladeshi children (SD(s) = 0.26 and 0.42, *P(s)*<0.07 [64, 65]; *P*>0.05 [66]). Additional studies suggest that the cognitive benefits of education are not specifically related to the mother, rather education attained by either parent can be a positive predictor (*P(s)*<0.05 [30, 41, 67, 68]; *P*<0.10 [69]; *P*_*univ*_<0.05 [58]; *P(s)*>0.10 [61, 63]).

Family assets (e.g., car, real estate, household items) assessed in three studies (IP n = 3) were associated with higher cognitive scores by as much as 0.25–0.85 SD. Household items, such as televisions, positively influenced cognitive ability in two Bangladeshi cohorts independent of the effects of the EOI (i.e., low birth weight or water contaminants (*P*<0.05 [70]; *P*<0.10 [65]). Homeownership was also a positive predictor of cognitive ability in Italian school children, independent of mercury (Hg) exposure (*P* = 0.04) [71].

Occupational social class (or job prestige) was assessed in six studies (EOI n = 1; IP n = 5). A father’s job prestige was a positive indicator of cognitive ability in two Australian cohorts (i.e., 1.2–2.0 points per point change in prestige score) [63, 72, 73]. This association remained significant for cognitive scores measured at four and seven years old independent of effects associated with cognitive stimulation at home and maternal intelligence (*P(s)*<0.05) [72]. In a second Australian cohort, paternal job prestige was a positive predictor independent of breastfeeding (P = 0.08) [63]. Better parent job prestige predicted higher cognitive scores in three of four studies independent of their EOIs (i.e., atopy, diet, Hg) (unskilled vs. professional—B(s) = -11.0 to -1.8; *P(s)*<0.05 [20, 74, 75]; *P*>0.05 [48]).

#### Inherent Factors–Child

The majority of studies that included gender as an IP (n = 32) found that girls scored 2–5 points higher than boys independent of the effects associated with the EOI (i.e., socio-demographic determinants, metals, parent-child interactions, prenatal diet, air pollutants, breastfeeding, synthetic chemicals, sleep duration, and drugs) (*P(s)*<0.05 [22, 23, 26, 27, 29, 40, 48, 51, 53, 54, 56, 58, 63, 70, 75–84]; *P(s)*>0.05 [30, 38, 41, 43, 62, 66, 71, 85–87]). The opposite was true in two studies after accounting for the effects of the EOI (i.e., health and demographic risks, thyroid health) (*P(s)*<0.05) [88, 89]. Multiple analyses for one U.S. cohort investigating the effects of pesticides, air pollutants and material hardship provided mixed results [31–34, 36, 37].

Eleven studies evaluated the association between low birth weight (LBW) status (i.e., <2500 g) or extremes of LBW (i.e., <1500 g or very and <1000 g or extremely LBW) and cognitive development (EOI n = 7; IP n = 4). LBW status adversely affected cognitive development as early as infancy as evidenced in ten month old Bangladeshi infants (not LBW–B = 2.7, *P*<0.05) [70]. In a cohort of Indian schoolchildren, children born with LBW were at risk of below average scores or cognitive delay (OR(s) = 1.76, 4.46, *P*_*univ*_*(s)* = 0.10, 0.02) with risk of cognitive delay heightened for children born VLBW (OR = 5.27, *P*_*univ*_ = 0.01) [90]. The six studies that examined very or extremely LBW as the EOI also found that these BWs were associated with lower cognitive scores (i.e., 7.6–16.2 points) as compared to those born full term among cohorts of children ranging in age from 1.5 to eight years old (*P*<0.05 [91]; *P*_*univ*_*(s)*<0.05 [92–96]). Effects of LBW status were also detrimental in three studies independent of EOIs (i.e., dietary Hg [71, 75] or economic indicators and parenting quality [23]).

LBW as a result of intrauterine growth restriction during multiple gestation was also a negative predictor of cognitive score at two years old in an Israeli cohort of triplets [97]. The cognitive scores of triplets with a discordant birth weight (i.e., 15% lower than the heaviest triplet) was six points lower than their siblings (*P*<0.05) [97].

The impact of current height and/or head circumference on cognitive development was assessed in seven studies (EOI n = 1; IP n = 6). Height was positively associated with cognitive scores in a cohort of Mexican toddlers independent of prenatal Pb exposure (Z-score B = 2.87, *P* = 0.05) [98]. In three Bangladeshi cohorts of school children height and/or head circumference were positive predictors of cognitive ability independent of the effects of water contaminants (i.e., arsenic (As), Mn) (per centimeter—height SD(s) = 0.04–0.07; head circumference SD(s) = 0.08–0.30; *P(s)*<0.05 [64, 65, 99, 100]). In two cross-sectional studies, height was not associated with cognitive scores after accounting for effects associated with the same contaminants (*P(s)*>0.05) [39, 47]. In a UK cohort assessing different growth parameters (e.g., height, head circumference, weight, BMI), these measures were correlated with better cognitive scores at seven years old only in those born very preterm (*P*_*univ*_*(s)*<0.05); whereas there was no correlation in full term children [101].

Three studies examined the impact of physical growth over time on cognitive development as the EOI. Growth (i.e., weight and height) during the first year of life and subsequent growth until five years old was associated with increased cognitive scores at six years old in a Belarusian cohort (difference in weight and height Z-score–B(s) = 0.29–0.77 and 0.57–0.84, *P(s)*<0.05) [102]. One UK cohort evaluated gains in height, weight and head circumference in multiple analyses. Head growth during the first year of life was positively associated with cognitive ability at four and eight years old, independent of later growth (Z-score difference B(s) = 1.56–1.97, *P(s)*<0.05) [103]. Increases in cognitive score were seen at eight years old with weight gain specifically between birth and two months (per SD difference in weight–B = 0.84, *P*<0.05) [55].

Fetal growth (i.e., head diameter, chest circumference and leg length) and its influence on cognitive ability was evaluated in a Scandinavian cohort as the EOI [49]. Fetal size at each trimester was a positive predictor of cognitive ability at 13 months old (Z-score difference per trimester B(s) = 1.35, 1.93 and 2.12, *P(s)*<0.05).

Ten studies evaluated the effect of preterm birth on cognitive development (EOI n = 8; IP n = 2). In general, results associated with any preterm birth (i.e., less than 37 weeks gestation) were inconsistent (*P*<0.05 [104], *P*_*univ*_*<*0.05 [105]; *P(s)*>0.05 [58, 60]. Any negative effects associated with preterm birth became evident when the severity of preterm birth was examined.

Moderate to late preterm birth (i.e., 32 to < 37 weeks) was a negative predictor of cognitive ability in seven year old Dutch children (B = 2.7, *P* = 0.03) [106]. However, the adverse effects may be restricted to girls in the Dutch cohort as suggested after stratified analysis [106]. In a U.S. cohort, preterm birth at this stage increased the risk of receiving a below average score at two years old as compared to full term birth (OR = 2.26, *P*_*univ*_<0.001) [107]. On the other hand, in a UK cohort, births in the moderate to late preterm stage were not associated with cognitive ability measured at eight years old even before adjusting for covariates (*P*_*univ*_ = 0.14) [108].

The negative effects associated with preterm birth were more consistent in studies that excluded children born late preterm (34 to <37 weeks). One study showed that five year old children born very or extremely preterm (≤32 weeks) were at risk of significant cognitive delay (OR = 3.78, *P*_*univ*_<0.001) [109]. Risk of cognitive delay was enhanced as gestation time decreased in this cohort, as well as in two other studies that evaluated extreme preterm birth (≤ 28 weeks) (OR(s) = 32, 23, *P*_*univ*_*(s)*<0.001) [110, 111]).

Severity of sleep disordered breathing (SDB) and/or symptoms (i.e., ranging from loud, frequent snoring to obstructive sleep apnea) in children between three and twelve years old was evaluated as the EOI in nine studies. Severity of SDB or symptoms observed in sleep studies or reported by parents was negatively associated with cognitive measures in the majority of studies (*P(s)*<0.05 [112, 113]; *P* = 0.06 [114]; *P*_*univ*_*(s)*<0.05 [115–117]; P>0.05 [118]; P_univ_>0.05 [119]). Frequent snoring symptoms in SDB diagnosed children regardless of SDB severity was also an indicator of lower cognitive scores (*P*_*univ*_*(s)*<0.05 [114, 116, 117, 120]). Two additional studies evaluating sleep-related patterns as the EOI found that daytime sleepiness was also a negative predictor of cognitive scores (*P*<0.05 [121];*P*_*univ*_<0.05 [122]).

#### Inherent Factors–Parents

Maternal IQ was included in the analyses of 28 studies evaluating cognitive development (EOI n = 2; IP n = 26). IQ was a positive predictor of cognitive development in Australian children assessed from two to 11–13 years of age independent of the effects of cognitive stimulation at home and occupational social class (per 10 IQ points—B(s) = 0.29–0.48, *P(s)*<0.05) [72]. In a Spanish cohort, higher maternal intelligence (i.e., tertile 3 vs. 1) appeared to impart a protective effect on early cognitive development only for children with mothers belonging to a lower occupational class after accounting for the effects of maternal mental health (B = 7.9, interaction *P*<0.05) [123]. No difference in association was seen based on maternal education [123]. In a second Spanish cohort, both maternal and paternal IQ were positive predictors of early cognitive development independent of each other and breastfeeding (per maternal IQ—B(s) = 0.02; *P(s)*<0.05) [61]. IQ was a positive predictor of children’s cognitive ability in 20 studies independent of the EOI (i.e., synthetic chemicals, drugs, socioeconomic determinants, genetics, parent-child interaction, maternal health, metals, diet, water and air pollutants, and pesticides) (*P(s)*<0.05 [20, 23, 24, 26–30, 37, 60, 64, 78, 79, 81, 84–86, 89, 100, 124–128]; *P(s)*>0.05 [65, 82, 83, 87]).

Signs of thyroid dysfunction during pregnancy associated with low free or total thyroxine (FT4 orTT4) in women without thyroid disease were evaluated in four studies (EOI n = 4). Low TT4 levels (i.e., <2.5^th^ percentile) assessed at 16–20 weeks gestation were negative predictors of early cognitive development in a cohort from China (B = -9.3, OR (cognitive score<100) = 12.98, *P(s)*<0.05) [129]. Two studies found that low FT4 levels (i.e., <10^th^ percentile) during early pregnancy (i.e., ≤20 weeks gestation) were predictive of lower cognitive scores measured in Spanish and Dutch toddlers (B(s) = -2.2 and -8, *P(s)*<0.05) [130, 131]. Conversely, a U.S. study found that low levels of FT4 (i.e., <3^rd^ percentile) specifically during the second trimester were not associated with cognitive ability in children of similar age [132].

Pre-pregnancy BMI was examined in four studies (EOI n = 1; IP n = 3). In a U.S. cohort, children born to underweight or severely obese mothers were at increased risk of below average scores as compared to those born to women with healthy weights (RR(s) = 1.36 and 1.38; *P*<0.05) [133]. In a Dutch cohort, the effect of pre-pregnancy BMI on cognitive ability at seven years old remained significant after accounting for the effects of the child’s BMI at 4 years old (per kg/m^2^- B_univ_ = -0.66, B = -0.62) [60]. This significance did not hold when BMI at 7 years old was included in the regression model [60]. Adverse effects were also associated with higher BMIs in two studies independent of prenatal Hg exposure in Spanish toddlers [75] and diet patterns in eight year old UK children [20].

Four studies included multiple gestation in their analysis of cognitive development (EOI n = 1; IP n = 3). Twin gestation was associated with lower cognitive scores independent of socio-demographic determinants in Greek toddlers [58] and independent of diet in UK schoolchildren [20] (B = -7.82 and -2.47, respectively; *P(s*)<0.05). Multiple gestation, in general, adversely affected cognitive development by two years of age in an Israeli cohort (β = -0.22, *P*<0.05) [97]. In fact, the cognitive scores of triplets at multiple assessments were lower than both that of twins and singletons (vs. singleton and twins–B(s) = -7.3 to -4.1 and -6.0 to -2.5, *P(s)*<0.05) [97]. Multiple gestation was not a significant predictor in a Scottish study evaluating the effects of thyroid health on cognitive scores by 5.5 years old [83].

Prenatal depression was evaluated in three studies (EOI n = 2; IP n = 1), one of which also evaluated the cumulative effect of maternal depression from pregnancy through childhood [134]. Exposure to mothers at high risk of prenatal depression adversely affected cognitive development as seen in three studies which noted a decrease in cognitive scores in Greek toddlers (B = -5.45, *P*<0.05) [135] and Canadian preschoolers (B = -5.1, *P*_*univ*_<0.05) [125]. High risk of prenatal depression was not a significant predictor of cognitive scores in eight year old UK children after adjusting for any postnatal depression [134]. A score of cumulative depressive symptoms, on the other hand, was not predictive of early cognitive development in the Canadian or Greek cohorts (*P*>0.05 [135]; *P*_*univ*_*(s)*>0.05 [125]).

Post-partum depression during infancy was evaluated in five studies (EOI n = 4; IP n = 1). Children born to mothers at higher risk for post-partum depression had lower cognitive scores as measured in the Greek cohort (B = -5.64, *P*<0.05) [135] and the UK cohort (B = -2.4, *P*_*univ*_<0.05) [134]. A third study found no association between risk of depression during this period and cognitive ability in an Australian cohort [136]. A score for cumulative symptoms of postnatal depression assessed two months after birth was also negatively associated with cognitive development in the Greek cohort (B = -0.33, *P*<0.05) [135] and in two year old French children (B = -0.85, *P* = 0.07) [57]. Conversely, depressive symptom scores did not appear to be associated with cognitive development in a U.S. cohort after accounting for the effects of mother-child interactions (*P*>0.05) [27].

Four studies suggest that high levels of prenatal stress assessed using an assessment tool and/or measuring cortisol levels, a biomarker of stress, may be predictive of lower cognitive scores (EOI n = 4). An increased risk of lower cognitive scores (≤25^th^ percentile) was noted in Dutch infants whose mothers experienced high stress due to everyday problems, specifically during early pregnancy, independent of postnatal stress and depression (OR = 1.1, *P*<0.05) [137]. Adverse effects on cognitive scores due to high stress levels were also found in a New Zealand cohort of pre-school children (B = -3.4, *P<*0.01) [138]. Similar results were presented in a third study of UK toddlers in which prenatal stress was a negative predictor independent of cortisol and postnatal stress (index score—B = -2.60, *P<*0.001) [43].

The effects of prenatal cortisol were evaluated in the Dutch and UK cohorts, as well as in a U.S. study. The studies yielded inconsistent results possibly due to timing of exposure. For the UK cohort, cortisol measures only during the first half of pregnancy were negatively associated with cognitive development between 1 and 2 years old (ln (nmol/L)—B = -9.45, *P*<0.05) [43]. In Dutch infants, the period of sensitivity to the negative effects of cortisol exposure occurred during late pregnancy (tertile 3 vs. 1 –B = 5.0, *P*<0.05) [137]. The U.S study revealed a difference in effect across pregnancy where cortisol levels during early pregnancy had an adverse effect (per μg/dL–β = -0.23) and levels during late pregnancy enhanced early cognitive development (β = 0.17; *P(s)*<0.05) [139].

#### Breastfeeding

Exposure to breastfeeding was examined in eleven studies (EOI n = 9; IP n = 2). The association between breastfeeding and cognitive ability was explored using three different types of measures (i.e., ever-exposed, duration and intensity of exposure).

Two studies evaluated ever being breastfed as an IP of cognitive development. Breastfeeding seemed to be associated with higher cognitive scores among a Mexican cohort in which scores were measured periodically over the child’s first year of life. The beneficial effects associated with breastfeeding were not apparent as early as the first month of life (*P*_*univ*_>0.05) [140]. However, breastfeeding over a child’s first year of life was a positive predictor of cognitive development from one to twelve months old independent of prenatal pesticide exposure (B = 1.14, *P* = 0.03) [141]. These effects were also evident in a UK study examining the association between physical growth and cognitive scores measured at four years old (B = 3.4, *P*< 0.01) [56]).

Duration of breastfeeding was included in eight independent studies and one multi-cohort study (EOI n = 5; IP n = 4). Together, these studies suggest that a minimum of four to six months of breastfeeding may benefit cognitive development. Two distinct Spanish cohorts included in a multi-cohort study examined the impact of breastfeeding duration on cognitive ability. Four to five months of breastfeeding (versus 2 weeks) was associated with a 10.7 point increase in cognitive ability at one year old in the first cohort [142]. In the second cohort, each month of breastfeeding was associated with a 0.56 point increase in cognitive score at four years old after adjusting for effects associated with atopy (*P* = 0.052) [74]. In an analysis that included both cohorts, a minimum of five months of breastfeeding was associated with a 7.7 point increase at four years old as compared to those children breastfed for less than one month (*P*<0.05), with a stronger protective effect seen in children prenatally exposed to high levels of DDT (>20 ng/mL, B = 13.04, *P*<0.05); thus counteracting any adverse effects associated with DDT [143]). Similar durations of breastfeeding (i.e., four to six months) were positively associated with children from four additional cohorts from this study (*P*_*univ*_<0.05 [75], *P*_*univ*_>0.05 [144]). Similarly, a second Spanish study found that a minimum of four months of breastfeeding (versus >0–4 months) was associated with increases in cognitive scores at 18 [145] and 24 months old [61] (B(s) = 4.6 and 4.3, *P(s)*<0.05). In a Polish cohort, breastfeeding for a minimum of 6 months was beneficial in regards to cognitive development even with inclusion of childhood exposure to mold (B = 4.0, *P*<0.05) [44]. Duration of breastfeeding was a positive predictor in one of two studies evaluating cognitive development in preschool children (per week–B = 0.29, *P*<0.05 [48], *P*>0.05 [63]). Preliminary analysis from both studies found that a minimum of five to six months of exposure was positively associated with cognitive scores (*P*_*univ*_*(s)*<0.05) [48, 63]. Breastfeeding duration was a significant predictor in one study after accounting for the effects of the child’s BMI (per month—B = 0.93, *P*<0.05) [60]; unlike, in studies evaluating thyroid health or pesticides [83, 146].

A pooled analysis study suggested that economic measures at the national level may influence the effect of breastfeeding (heterogeneity *P* = 0.09) [147]. The study showed duration of breastfeeding (i.e., 0-<1, 1-<3, 3-<6 and 6 months) having a stronger effect on cognitive ability in four year old Brazilian children (B = 1.97) as compared to eight year old UK children (B = 0.97, *P(s)<*0.001), with significant increases in cognitive scores associated with a minimum of three months duration for both groups (*P(s)<*0.05) [147]. In a second analysis of the UK cohort, the beneficial effects of breastfeeding by one month were also independent of later childhood diet (*P(s)*≤0.051) [20].

The UK, Spanish multi-cohort, Polish studies, and an Italian study also evaluated the effect of exclusivity or intensity of breastfeeding (EOI n = 4). Two studies found that a higher proportion of breastfeeding in a child’s diet during the first year of a child’s life can significantly improve cognitive scores as early as fourteen months old as seen in the Spanish cohort [148] and as late as eight years old as seen in the UK cohort [149] (*P(s)*<0.05). In fact, the beneficial effects of exclusive breastfeeding for six months or longer superseded those of complementary feeding in the Polish and Spanish cohorts (B(s) = 2.5–3.5, *P(s)*<0.05) [54, 148] and duration of exclusivity in an Italian cohort (per week, B = 0.04, *P* = 0.09) [150] may account for variations in the beneficial effects of breastfeeding.

Long Chain Polyunsaturated Fatty Acids (LC-PUFAs)—The benefits of breastfeeding may be associated with essential nutrients present in breast milk as suggested by the Spanish cohort study [148]. Maternal sources of 11 LC-PUFAs and their association with cognitive development were examined in five studies (EOI n = 4; IP n = 1). LC-PUFAs obtained through childhood diet were also examined in two studies (EOI n = 2).

The effect of total and/or individual omega-3 and omega-6 LC-PUFAs was examined in six studies (EOI n = 5; IP n = 1). Spanish toddlers exclusively breastfed for longer duration (i.e., >4 months) benefitted from higher levels of omega-3 LC-PUFAs as measured in the mother’s first milk (B = 4.85, *P*<0.05) as compared to those infants never exclusively breastfed and exposed to lower LC-PUFA levels [148]. Total omega-3 LC-PUFAs were not predictive of cognitive ability in Seychelles toddlers after accounting for the effects of Hg vapor exposure [82].

Docosahexaenoic acid (DHA), an omega-3 LC-PUFA, was a positive predictor in three of five studies. Two studies found that DHA (per μg/mL) measured from postnatal sources (i.e., breast milk [48] or child’s diet [151]) was positively associated with cognitive ability in Swedish (B = 0.92, *P<* 0.01) [48] and Egyptian children (B = 0.52, *P*_*univ*_<0.05) [151]. Additionally, a study of Inuit infants found that prenatal DHA levels were associated with higher cognitive scores (per μg/mL-B = 0.6, *P<*0.05) [152]. DHA was not associated with cognitive development in Dutch school children [153] or Spanish toddlers [148] (*P(s)*>0.05).

The beneficial effects of LC-PUFAs and breastfeeding may be attributed to mechanisms known to regulate LC-PUFA metabolism, as suggested by additional analyses in a Swedish study [48]. An analysis of the Spanish multi-cohort that included a cohort of toddlers and a cohort of four year olds assessed fatty acid desaturases (FADS), elongase-2 (ELOVL2) and ELOVL5 genes [154]. Variants in ELOVL and FADS in both the mother and child were identified as being associated with cognitive development (*P(s)*<0.05). In fact, the beneficial effects of ever being breastfed were only seen in children with specific polymorphisms for these enzymes resulting in higher cognitive scores by as much as five to nine points (*P(s)*<0.05) [154].

#### Other Dietary/Nutrition Factors

Two UK studies examined the effect of dietary patterns on cognitive development (EOI n = 2). Early dietary patterns for infants/toddlers consisting of higher amounts of fruits and vegetables, homemade foods and breast milk were positively associated with cognitive scores for both cohorts (*P(s)*<0.01) [149, 155]. Early diets that included processed foods and snacks were negative predictors of cognitive ability in the older cohort (*P*<0.05 [20, 149]; *P*>0.05 [155]). The effects of diet patterns after toddlerhood (i.e., 3, 4, 7 and 8 years old) on cognitive scores varied in this cohort (*P(s)*<0.05) [156].

Six studies assessed the impact of childhood iron levels through the measurement of different biomarkers (i.e., ferritin, transferrin, hemoglobin) (EOI n = 1; IP n = 5). Early Fe deficiency in children adversely affected cognitive scores measured at four years old Fe levels independent of any effects associated with prenatal drug use and Pb (B = -8.1, *P*<0.05) [87]. Childhood Fe levels were a positive predictor in studies primarily evaluating the effect of environmental contaminants (i.e., As, Mn, Pb) on cognitive development (*P(s)*<0.05 [38, 100, 157]; *P(s)*>0.05 [40, 47].

Prenatal folate intake and/or methylenetetrahydrofolate (MTHFR) genotype were evaluated in four studies as the EOI(s). MTHFR polymorphisms (e.g., 677, 1298) are associated with reduced enzymatic activity resulting in slower folate metabolism. Deficient folate intake through diet posed a risk for children whose mothers carried the homozygous variant of MTHFR 677 (i.e., TT) (B = -1.8, *P*<0.05) [158]. In a second Mexican cohort, maternal MTHFR 677TT was associated with lower cognitive scores (B = -3.52, *P* = 0.004) after accounting for the effects of low folate intake, which was not a significant predictor (*P*>0.05) [127]. Haplotype analysis of maternal MTHFR 677 and 1298 polymorphisms showed that only children of mothers with both variants have lower scores as compared to mothers with neither variant (*P*<0.05). No associations were seen with fetal MTHFR in this cohort. Alternatively, for mothers whose diets already provide high folate levels (e.g., Mediterranean), additional folate supplementation had no added benefit to early cognitive ability in Greek [159] or Spanish [160] cohorts (*P(s)*>0.05).

#### Lifestyle Behaviors

Prenatal alcohol exposure was examined in fourteen studies (EOI n = 7; IP n = 7). Most studies found no association with cognitive development. However, four studies that examined level of prenatal alcohol consumption (i.e., low, moderate, heavy) consistently found that moderate (0.5 to ≤1 drink/day) to higher exposures adversely affected cognitive development in school-aged children. A U.S. study showed a negative association between moderate to high amounts of alcohol exposure and cognitive ability in preschool-aged black children (at risk vs. < at risk-levels—β = -0.24, *P<*0.01) [161]. No difference in cognitive scores was seen in eight year old UK children whose mothers reported moderate to high consumption of alcohol during pregnancy as compared to no alcohol exposure [20]. However, further analysis indicated that some children were vulnerable to the effects of prenatal alcohol exposure at this level depending on the alcohol metabolism enzyme genotypes of the mother and child (per allele—B(s) = -1.27,-1.95, *P(s)*<0.05) [162]. The negative effects on cognitive ability were limited to higher or more frequent drinking behaviors in a Dutch study, which found a risk of significant cognitive delay associated with greater than eight drinks per week as compared to no exposure (OR = 4.6, *P*<0.05) [163, 164]. In contrast, the effects of moderate to high alcohol consumption had no significant effect on cognitive scores in an adolescent cohort from Australia [165].

Fathers or male figures in the household with alcohol-related problems or dependence was a negative predictor in two cohorts (EOI n = 1; IP n = 1). A father diagnosed with alcohol dependence was associated with lower cognitive scores in school children from India (B = -9.0, *P*_uandj_<0.05) [166]. A U.S. study found that in addition to the negative effects associated with prenatal marijuana exposure, children living with a male with alcohol-related problems scored 3.2 points lower than those without this exposure *(P<0*.*05)* [28].

### Social Environment

Both direct and indirect interactions within the social environment can foster cognitive development. These include not only stressors within the home, but more distal stressors within the school and the greater community, which may have a similar or even an additive impact on children’s health. Interactions which stimulate cognitive development can serve as a protective measure against other exposures which may be negatively associated with child development. Alternatively, negative social interactions may exert a negative influence itself or enhance the adverse effects of other variables. Elements within the postnatal social environment have psychosocial implications which can impact their performance at the time of cognitive assessment. The social environment of the mother during pregnancy has a direct psychosocial impact on the mother, but an overwhelmingly biological impact on the fetus. Because of this, this section only includes factors related to a child’s postnatal social environment.

#### Family Structure

Two studies assessed how alternative care for children can affect cognitive development (EOI n = 2). Preliminary analyses for both studies found children currently [167] or previously [168] in foster or long-term institutional care had significantly lower cognitive scores as compared to children never fostered or adopted (foster care d(s) = -0.72 [167] and -0.53 [168]; institutional care d = -1.08 [168]) (*P*_*univ*_*(s)*<0.05).

The association between siblings and cognitive development was evaluated in five studies (EOI n = 1; IP n = 4). In a Greek cohort, toddlers with older siblings scored 2.9 points lower than children without siblings after accounting for effects associated with demographic and biological measures (*P*<0.05) [58]. The number of older siblings was a negative predictor of early cognitive ability in two U.S. cohorts after accounting for the effects of maternal employment (B = -2.06) [27] or prenatal cocaine exposure (B = -1.48) [25] (*P(s)*<0.05). One of two studies found that having more than one sibling was a negative predictor of cognitive ability independent of the EOI (i.e., childhood diet, B = -1.93, *P* = 0.07 [20]; breastfeeding, *P*>0.10 [61]).

Two studies accounted for the influence that changes in family structure can have on cognitive development (EOI n = 1; IP n = 1). In a UK study, mothers with a history of unstable relationships with partners was a negative predictor in a study primarily examining the effects of early child care in toddlers (B = -6.6, *P*<0.05) [22]. Preliminary analysis in a second study showed no significant differences in cognitive scores among children who lived in single parent or stable two parent households in a study of Belarusian children (*P*_*univ*_>0.05) [169]. However, introduction to new family members (i.e., step-family) did negatively impact cognitive ability in this cohort (B = -1.3, *P*_*univ*_<0.05) [169].

#### Parenting Behaviors

Cognitive stimulation from parents (i.e., different tasks including book reading, singing and/or playing) was evaluated in two U.S. studies (EOI n = 1; IP n = 1). In a cohort representative of children across the U.S., frequency of a mother reading to a child daily over time (i.e., 14–36 months) was associated with better cognitive scores at two and three years old (B = 3.4, *P*<0.05) [26]. Either parent engaging their child in play was another form of stimulation that was positively correlated with cognitive scores at three years old (*P*_*univ*_*(s)*<0.01) [67]. The benefits of paternal cognitive stimulation through activities including book reading significantly benefitted early cognitive development in a cohort of low income children (β = 0.14, P< 0.001) [88].

Opportunity for cognitive stimulation present within the child’s home rather than specific tasks was included in 23 studies (EOI n = 2; IP n = 21). The home observation measurement environment (HOME) inventory was the most common tool used to measure both the quality and quantity of opportunities for cognitive stimulation within the home. In an analysis that included breastfeeding and socioeconomic status (SES), cognitive stimulation was the strongest predictor of cognitive ability in a four year old Australian cohort (per SD in Home Screening Questionnaire score- B = 1.8, *P*<0.05) [63]. The beneficial effects of cognitive stimulation at home were seen as early as six months old in a Mexican-American cohort, in which this variable was a stronger predictor of cognitive ability as compared to other parenting behaviors assessed in the same analysis (HOME score—B = 0.8, *P* = 0.10) [170]. A second Australian study evaluating cognitive development from two to 11–13 years of age suggested that early cognitive stimulation at home may also be predictive of later cognitive measures. This exposure measured at three years old had a stronger influence on cognitive scores at all ages of assessment as compared to other significant predictors in the same analysis (i.e., SES, maternal intelligence) (HOME score—B(s) = 0.4–0.9, *P(s)*<0.05) [72]. Cognitive stimulation at home was also a positive predictor in UK toddlers independent of any cognitive stimulation received at child care (higher HOME score—B = 3.4, *P<*0.01) [22]. Multiple analyses of a U.S. cohort consistently showed that cognitive stimulation was a positive predictor of cognitive development over time (i.e., one to five years old) regardless of the EOI (i.e., air pollutants, pesticides) (HOME score—B(s) = 0.33–0.59, *P(s)<*0.03 [32–34, 37]), with the exception of one study which measured cognitive ability at seven years old (*P*>0.05) [36]. Similar results were found in studies independent of the EOI (i.e., prenatal exposure to drugs, pesticides, synthetic chemicals, Pb, As, Hg, LBW, depression, employment and diet) (*P(s)*<0.05 [25, 27–29, 64, 70, 76, 81, 82, 84, 86, 89, 124, 136, 141]; *P(s)*<0.10 [20, 59, 87]).

The overall quality of the interactions between a mother and child (e.g., sensitivity, responsiveness) was the EOI in four U.S. studies. Early measures of parenting quality were a positive predictor of cognitive development in three cohorts of toddlers independent of the effects of a child’s own persistence (β = 0.30, *P*<0.05) [35], other parent behaviors (β = 0.0.06, *P*<0.05) [88], or economic indicators (β = 0.17, *P*<0.05) [23]. Current measures of quality of interaction were not associated with cognitive development in the first cohort [35] or in a cohort of Mexican-American infants after adjusting for HOME [170] (*P(s)*>0.05).

Supportive behavior (e.g., warmth, nurturing, and positive regard) were assessed in four U.S. studies (EOI n = 4). Early displays of support by mothers and fathers were positively associated with early cognitive ability independent of other behaviors (β(s) = 0.20–0.29, *P(s)*<0.05) [26, 88]. Maternal nurturing (r = 0.36) [170], communication (β = 0.43) [171], and positive regard from both parents (r(s) = 0.14–0.29) [67] were also positive correlates of early cognitive development (*P*<0.05, *P*_*univ*_*(s)*<0.05).

#### Social Support

Two studies evaluated the association between social support from family and friends and cognitive development (EOI n = 1; IP n = 1). An Australian study found that low levels of social support during pregnancy were negatively associated with cognitive scores for 3–4 year old children (1^st^ vs. 4^th^ quartile—B = -3.1, *P* = 0.03) [138]. Social support at the time of cognitive assessment also imparted a protective effect in children born to mothers with high levels of stress (B = -0.6, *P*>0.05) as compared to those with lower levels of support (B = -4.1, *P* = 0.08) [138]. The level of social support was also a positive predictor of cognitive scores for a U.S. cohort of six year old children independent of the effects associated with prenatal marijuana exposure (B = 0.94, *P*<0.001) [28].

#### Child Care or School

Type of care (e.g., group or center, individual care) was assessed in two studies (EOI n = 1; IP n = 1). Child care at a center or group setting imparted a slight, yet significant, benefit to cognitive development in a UK cohort of toddlers independent of the quality of child care (B = 0.17, *P<*0.01); while individual child care had no effect [22]. Similarly, in a U.S. study evaluating the effect of maternal work schedules, center care was the only type (i.e., center, family daycare, relative, non-relative) of child care positively associated with cognitive scores measured at two years old (B = 0.81, *P<*0.01) [27].

A composite measure of child care quality (i.e., stimulation, sensitivity, adult child ratio) was assessed in two studies (EOI n = 1; IP n = 1). Better quality child care was associated with an average increase of 3.3 points in cognitive scores in a UK cohort of toddlers (β = 0.19, *P*<0.01) [22]. Increases in cognitive scores were also seen in a U.S. cohort of toddlers enrolled in child care rated as above average (β = 0.09, P<0.01) [27]. A second UK study found that when qualities were treated as individual predictors within the same analyses, only cognitive stimulation provided the most benefit for infants (β = 0.38, *P*<0.05) [69]. However, the effect associated with cognitive stimulation was strongest if it was provided by caregivers rated higher for sensitivity (interaction *P* = 0.055) [69].

School attendance was an IP in two Bangladeshi studies examining the effects of water contaminants on cognitive development. Attendance rate (i.e., days/week or total months) was a positive predictor of cognitive scores even after adjusting for the effects of As and/or Mn (β(s) = 0.11 and 0.5, *P*(s)<0.05) [64, 100].

#### Built and Natural Environments

The physical environment is made up of both natural and built characteristics which may have an effect on child development. Chemicals of concern present in the physical environment (i.e., soil, air, water) come from both natural and man-made sources. Regulations have been established for some, but not all, chemicals of concern due to adverse human health effects including those related to cognitive development. Non-chemical aspects of the natural and built environments garner attention because they can introduce or modify non-chemical and chemical stressors, thus, altering a child’s exposure.

#### Arsenic (As)

Seven studies examined the association between As exposure and cognitive ability in school children (EOI n = 7). Children in these studies were from countries with drinking waters known to have high As levels far exceeding the recommended U.S. standards (i.e., 10 μg/L [172]). Results from cohort studies of Bangladeshi, Chinese and Mexican children with co-exposures to other elements such as fluoride and Mn suggested that concurrent As levels in drinking water were a negative predictor of cognitive ability (0.05–0.41 per log(μg/L), *P(s)*<0.05 [47, 64, 65]; with cognitive scores dropping by as much as 0.30 to 0.54 SD in children exposed to levels greater than 176 μg/L (vs. <10)(*P(s)*<0.05 [65], *P*_*univ*_<0.05 [173]). As measured in urine or blood was also predictive of cognitive scores (*P(s)*<0.05 [100]; *P(s)*<0.10 [47, 65]). Evidence from a separate cohort of Bangladeshi children suggested that both prenatal and postnatal As levels adversely affected cognitive development measured at five years old [174, 175]. The effects associated with cognitive scores at five years old were strongest for girls and specific to As exposures during late pregnancy and at the age of cognitive assessment (0.15–0.21 SD’s per log(μg/L), interaction *P(s)*≤0.06) and late pregnancy (interaction *P* = 0.06) [175]. Conversely, one study found that neither period of As exposure was predictive of cognitive scores measured in Indian children ranging in age from 5–15 years old (*P(s)*>0.05) [176].

#### Fluoride (F)

Five cross-sectional studies of school-aged children (i.e., 6–12 years old) from countries (e.g., India, Iran, Mexico and China) with elevated fluoride levels in groundwater (i.e., >4 mg/L per U.S. recommendations [177]) suggest that fluoride adversely affects cognitive development (EOI n = 5). Levels greater than 4 mg/L were associated with cognitive scores 0.3–0.6 SDs lower than those exposed to levels below 1–2 μg/L [173, 178]. In fact, even levels below the recommended maximum level were found to be associated with lower cognitive scores or risk of below average scores in the Iran cohort (>1 mg/L, OR = 1.75) [178] and an Indian cohort (≥1.5 mg/L) [179] (*P*_*univ*_*(s)*<0.05). Fluorosis, an indicator of high fluoride exposure, was also associated with low cognitive scores (<80) in a second Indian cohort (OR = 2.91, *P*_*univ*_<0.002) with stronger effects in girls (*P*_*univ*_ = 0.003) [180]. Fluoride also remained a predictor of cognitive development in Mexican children in a study examining co-exposure to As (0.68 SD per log (mg/L), *P<*0.001) [47].

#### Lead (Pb)

Seventeen studies evaluated the effect of Pb on cognitive development (EOI n = 13; IP n = 4). Ten studies restricted their cohort samples to children with blood Pb (BPb) levels less than or equal to 10 μg/dL to understand the effects associated with lower exposure levels. Historically, research has focused on the effect of Pb on cognitive function with moderate to high doses of Pb [181].

Thirteen studies assessed childhood Pb exposure (EOI n = 10; IP n = 3). One additional study assessed gene-environment interactions associated with Pb. A study of black U.S. children found differences in cognitive scores at seven years old between children with BPb levels above and below 5 μg/dL (*P*<0.05) and suggested the possibility of adverse effects with levels as low as 3 μg/dL (*P*< 0.10) [182]. A second study restricted to U.S. school children with BPb levels under 10 μg/dL also found differences in effect on cognitive ability associated with 5–10 μg/dL BPb (B = -6.04, *P* = 0.01) as compared to 1–2 μg/dL BPb (B = -0.12, *P* = 0.94) [24]. Similar results were yielded in a U.S. cohort that found declines in cognitive scores in six year olds with BPb levels under 10 μg/dL (5–10 μg/dL vs. <5 μg/dL -B = -3.7, *P* = 0.10 [183]; per μg/dL–B = -1.58, P<0.05 [184]). In two U.S. cohorts that included children with prenatal drug exposure, Pb exposure adversely influenced cognitive ability at four years old (*P(s)*<0.05) [87, 185], but this association did not hold true when one cohort was restricted to children with BPb levels under 10 μg/dL (*P*_*univ*_ = 0.23) [185]. In a U.S. cohort with initial BPb levels greater than 20 μg/dL at two years old, concurrent Pb levels were a negative predictor of cognitive development measured until seven years of age independent of effects associated with previous Pb, Hg and Mn exposures (per μg/dL B(s) = -0.4 to -0.2, *P(s)*<0.05) [30, 186, 187].

These effects were not restricted to U.S. cohort studies as evidenced by a pooled analysis of cohorts from multiple countries (including [73, 85, 183]) [45] and a Mexican cohort of two year olds [79], which revealed steeper declines in cognitive scores with increasing Pb exposure in children with concurrent BPb levels under 7.5–10 μg/dL as compared to those with greater exposure (P<0.05). BPb levels were also predictors independent of the effects associated with Mn exposure as evidenced in this cohort of Mexican toddlers [188], as well as in older children from Italy [157] and Korea [41] (*P(s)*<0.05). Conversely, in a study primarily investigating the effects of Mn exposure in a second cohort of Mexican children, concurrent Pb exposure greater than 6 μg/dL was not associated with their cognitive ability [38]; with similar findings in two additional Mexican cohorts (*P(s)*>0.05) [47, 85]. However, further stratification of children by Mn exposure may show that those with higher Mn exposure may be more vulnerable to Pb toxicity as evidenced in two of the previous cohorts co-exposed to Pb and Mn (*P(s)*<0.05) [41, 188]. Similar vulnerabilities to concurrent Pb levels were also evident in Polish school children with specific polymorphisms (i.e., δ*-*aminolevulinic acid dehydratase (ALAD) and Vitamin D receptor (VDR)) whose products may modify Pb availability and toxicity (interaction *P(s)*≤0.02) [189]. There was no direct association between variants of ALAD or VDR and cognitive ability.

Evidence from seven studies suggested that prenatal exposure may adversely affect cognitive development possibly more so than concurrent postnatal exposure (EOI n = 5; IP n = 2). However, the exact timing of exposure in which the child is most susceptible during pregnancy is unclear. One study of Polish children with umbilical cord (fetal) BPb levels under 5 μg/dL suggested that fetal exposure to low BPb levels may adversely affect early cognitive development (per log(μg/dL)–B = -6.7, *P* = 0.02), with effects being more prominent in boys (interaction *P*<0.05) [53] and in those born to mothers without any college education (*P*_*univ*_<0.05) [52]. In a Mexican cohort of two year olds, fetal Pb exposure was negatively associated with cognitive scores even after accounting for effects associated with maternal and child MTHFR genotypes (per μg/dL–B(s) = 0.7, *P(s)*<0.05) [127]. Fetal Pb levels were not predictive of cognitive development in a U.S. cohort of two year old children after accounting for the effects of other contaminants and material hardship [31].

Studies which measured Pb in maternal blood gave more insight into the exact timing in which cognitive development is most sensitive to Pb toxicity. Maternal BPb levels during late pregnancy negatively influenced cognitive development in Mexican school children (per ln(μg/dL)–B = -4.1, *P<*0.01) [85]. Umbilical cord, early pregnancy and postnatal measures were not associated with cognitive ability at this age [85]. Results from a study of Korean infants suggested that cognitive development by as early as six months may also be sensitive to low maternal BPb levels (<10 μg/dL) during late pregnancy (*P* = 0.02), with children co-exposed to higher Cd levels (>1.51 μg/L) during this period being most vulnerable to these adverse effects (per log(μg/dL)–B = -12.1, *P<*0.01) [190].

Contrary to this, maternal BPb levels during late pregnancy did not influence cognitive development in two other studies which measured Pb exposures at multiple time points. Analyses of a Mexican cohort found that decreases in cognitive scores measured at two years old were specific to prenatal (i.e., first trimester) (per log_e_ (μg/dL)-B = -3.5) and postnatal (i.e., birth, 1, 2 years old) Pb exposure (*P(s)*<0.05) [98]. Stratification analysis for this cohort revealed that early postnatal Pb exposure (per μg/dL) may influence early cognitive development (i.e., 1 to 3 years old) specifically for children with BPb levels under 10 μg/dL (B = -1.04) [79], those born to mothers with low self-esteem (B = -0.31) [78], or those co-exposed to high Mn levels (B = -1.3) [188] (*P(s)*<0.05). In a Taiwanese cohort with BPb levels below 10 μg/dL, third trimester BPb was not associated with any period of cognitive development (i.e., 2–3, 5–6, or 8–9 years old) (*P(s)*>0.05) [59]. However, earlier postnatal measures rather than concurrent exposures were associated with cognitive measures at 5–6 and 8–9 years old (per ln (μg/dL)-B = -6.0, *P*<0.05) [59]. In a U.S. cohort of children with BPb levels over 20 μg/dL at baseline, both cumulative and concurrent postnatal measures were significant predictors (*P(s)*<0.05) [187]. In a second U.S. study, all postnatal exposure measures (i.e., early postnatal, concurrent, cumulative) were predictive of cognitive scores (*P(s)*<0.05) [183, 184].

#### Manganese (Mn)

Results from eight studies (EOI n = 7; IP n = 1) found that concurrent Mn levels measured in water, blood or hair negatively influenced cognitive development of school children from areas with exposure levels that exceeded the recommended U.S. guidelines (i.e., 50 μg/L in water or 5 mg/m^3^ in air [191]) (*P(s)*<0.05 [38, 39, 41, 65, 99, 192]; *P<*0.10 [100]; *P*>0.10 [64]). Studies have suggested that some groups may be more vulnerable to the effects of Mn. Within a Mexican cohort of school children, girls and younger children were more sensitive to the adverse effects associated with Mn (interaction *P(s)* = 0.06) [38].

Deficits in Mn exposure may also have an adverse effect on cognitive development over time as evidenced in a second cohort of Mexican children [40]. Children with Mn levels below 20.2 μg/L and above 28 μg/L had significantly lower cognitive scores between one and two years old as compared to those with moderate Mn exposure (*P*<0.05).

Three studies that examined co-exposures to Mn and other elements revealed how effects associated with Mn may be modified by another contaminant or vice versa. In co-exposure models for As and Mn measured in Bangladeshi children, As appeared to preclude any effects on cognitive scores associated with Mn (*P(s)*>0.05) [64, 65, 100]. Similar to Cd, elevated Mn levels may also enhance Pb toxicity as suggested in two studies examining cognitive ability in Korean infants and children from Mexico City with co-exposures to Pb and Mn [41, 188].

#### Indoor Nitrogen Dioxide (NO2)

Indoor NO_2_ was evaluated in two cohorts from the Spanish multi-cohort study (EOI n = 1; IP n = 1). Indoor NO_2_ had a negative effect on cognitive scores in a four year old cohort independent of any effects associated with atopy [74]. Among these children, those carrying the glutathione S transferase P1 (GSTP1) Ile105Val minor allele had less detoxification enzyme activity or efficiency and, as a result, were more susceptible to these effects (B = -3.36 per 10 μg/m^3^, interaction *P* = 0.04).

Similarly, use of domestic gas cookers, a source of indoor NO_2_, negatively influenced early cognitive development in a younger Spanish cohort (B = -2.5, *P*<0.05) [193]. Children with non-smoking mothers or in smoke-free homes were more vulnerable to these effects. However, older homes, urban neighborhoods and higher outdoor NO_2_ levels also enhanced these effects. Similar to the previous study [74], children carrying a minor allele(s) for GSTP1 Ile105Val were also more susceptible to the effects associated with gas cooking (B = -6.5, *P*<0.05). Whereas, higher prenatal fish and vegetable/fruit consumption and breast feeding greater than six months seemed to impart a protective effect.

#### Polycyclic Aromatic Hydrocarbons (PAHs)

Three studies evaluated the impact of prenatal PAH exposure on cognitive development (EOI n = 3). A longitudinal U.S. cohort study found that exposure to higher levels of PAHs (>2.26–4.16 ng/m^3^) resulted in a 5.7 point decrease by age three years (*P* = 0.02) [33] and a 4.3 point decrease at age five (*P*<0.01) [37] as compared to lower levels of exposure. In fact, there was a 2.89 times greater risk of below average scores at three years old with higher levels of PAH exposure (*P*<0.05) [33]. However, this association was no longer significant for the same cohort when additional pollutants and economic disparities were considered [31, 36]. The effects of prenatal PAH exposure were also associated with lower scores at five years old in a Polish cohort of children exposed to higher levels of PAHs (>17.96 ng/m^3^, B = -1.4, *P* = 0.04) [50]. A combined analysis of both cohorts to evaluate gene environment interactions found significant modification of PAH effect on cognitive score by selected variants in cytochrome P450 and GST genes with most of these interactions being unique to a specific race or ethnicity (i.e., Polish, Dominican or black) [21]. The negative effects of PAHs were confirmed in a third study where PAH exposure was estimated by measuring umbilical cord blood DNA adducts [126]. However, this negative effect was restricted to children whose mothers were exposed to ETS while pregnant (*P* = 0.02).

#### Polybrominated Diphenyl Ethers (PBDEs)

Three studies evaluated the sum effect of up to 14 different PBDEs (∑PBDE) either in blood or breast milk (EOI n = 3). Total PBDE exposure measured in blood were yielded adverse effects on cognitive score in two studies. Childhood and prenatal ∑PBDE_4_ exposure were associated with declines in cognitive scores (i.e., 4–5 points per log(ng/g)) at seven years old in a cohort of Mexican-American children (postnatal median = 84.4 ng/g lipid, *P*<0.05; prenatal median = 24.9, *P<*0.10) [194]. Risk of below average cognitive scores associated with prenatal ∑PBDE_11_ measured in cord blood may occur as early as infancy as shown in a Taiwanese cohort (>median (4.63 ng/g lipid), OR = 1.13, *P*<0.05) [195].

Conversely, no significant correlation was seen in the Taiwanese infant cohort with PBDE exposure through breast milk (∑PBDE_14_ median = 2.92) (*P*_*univ*_>0.05) [196]. Any potential negative association for PBDE exposure through breast milk in a cohort of Spanish toddlers was no longer apparent after adjustment for organochlorine pollutants (∑PBDE_7_ median = 3.50 ng/g lipid, *P* = 0.21) [197]. However, PBDE-209 was a negative predictor in both studies. There was evidence that PBDE-209 in breast milk, one of the main congeners present in the highest concentrations in both cohorts, potentially affected cognitive development as seen in the Taiwanese cohort (*P*<0.05) [196]. Duration and timing of PBDE-209 exposure may be an important factor to consider, as seen in the Spanish cohort in which a stronger association was seen in children breastfed longer than four months even after adjusting for additional pollutants (per log (ng/g) ->4 months, B = -3.48 *P*<0.05; ≤4 months, B = -1.07, *P*>0.05) [197].

Three additional studies examined the effects of individual PBDE congeners (EOI n = 3). Effects seen with prenatal ∑PBDE exposure may be specific to individual congeners including PBDE-15, -47, -85, -99, -100, -157 (*P(s)*<0.05) [195, 198]. Postnatal PBDE-47 exposure was also evaluated in an older Spanish cohort in which no associations were seen with cognitive scores measured at four years old (*P*>0.05) [199].

#### Phthalates

Three studies evaluated the association between phthalate exposure and cognitive development (EOI n = 3). Effects associated with prenatal phthalate exposure may be gender specific as suggested by one study evaluating early cognitive development in Korean infants [200]. Decrements in cognitive scores at six months old with increasing dibutyl and di-2-ethylhexyl phthalate (DBP and DEHP) exposure were greater for boys (per ln(μg/g creatinine) males-B(s) = -1.6 to -0.9, *P(s)*<0.05) as compared to girls (*P(s)*>0.05) [200]. However, the differential effects seen may be dependent on the metabolite measured. In a U.S. cohort, girls were more susceptible to any negative effects associated with mono-n-butyl phthalate, a metabolite of DBP, on cognitive development by three years old (*P<*0.01, interaction *P* = 0.054) [201]. Postnatal exposure to DEHP was negatively associated with cognitive scores in Korean school children (per ln (μg/g creatinine—B = -2.3, *P<*0.01) [202]. Adjustment for maternal intelligence weakened this association (*P*>0.55).

#### Pesticides

Studies examining the effects of pesticides (i.e., mirex [203, 204], chlorpyrifos [32, 36, 205, 206], dialkyl phosphate (DAP) [205, 207, 208], dichlorodiphenyltrichloroethane (DDT) [74, 141, 143, 146, 199, 209–211], hexachlorobenzene [204, 212]) as the EOI consistently found no association with cognitive development (S2 Table) with the exception of three studies that considered gene-environment interactions. Variants of paraoxonase-1 (PON1) and glutathione S transferases (GSTs) are detoxification enzymes known to detoxify organophosphate (OP) pesticides. The moderating effects of PON1 were examined in Mexican-American and urban U.S. cohorts. Mexican-American children homozygous for the minor allele PON1 R192Q, which is associated with reduced enzyme activity, and those with one or more minor alleles for PON1 C108T, which are associated with decreased enzyme levels, were more susceptible to the adverse effects of prenatal DAP on cognitive development at two years old (per log_10_(nmol/L)-B(s) = -7.4 to -3.4, *P(s)*<0.10) [213]. Conversely, in an urban cohort where both black and Hispanic infants were susceptible to DAP, those born to mothers with one or more major allele for PON1 R192Q were more susceptible to the effects of OP pesticides on cognitive scores at one year old (per log_10_(μmol/L)-B(s) = -4.9 to -4.5, *P(s)*<0.05) [214]. In a third study examining cognitive scores in a four year old Spanish cohort, children heterozygous for the minor allele, GSTP1 I105V, were more susceptible to the adverse effects of prenatal DDT exposure (per ng/mL -B = -9.4, *P* = 0.04, interaction p = 0.05) [215].

## Discussion

### Summary of Findings

**Fig 2:**
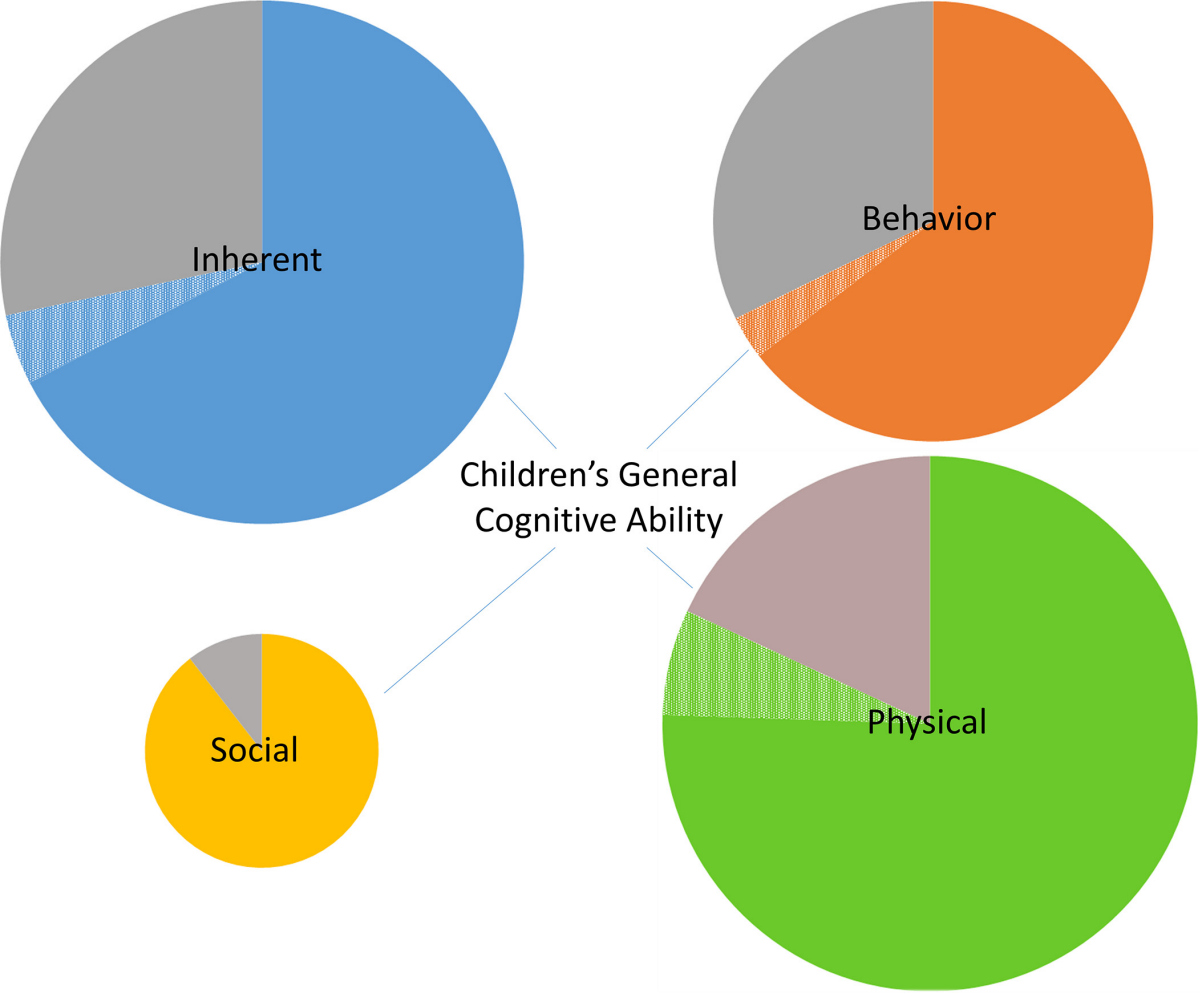
Factors Associated With General Cognitive Ability Grouped Into Four Domains: Inherent, Behavioral, Social Environment and Physical Environment. The size of the circles are in proportion to the total number of studies. The colored section in each circle represents the proportion of studies within each category that found a statistically significant association with general cognitive score (solid color–*P*<0.05; pattern – 0.05<*P*<0.10). This graphic is adapted from Strina et al. [282].

**Fig 3:**
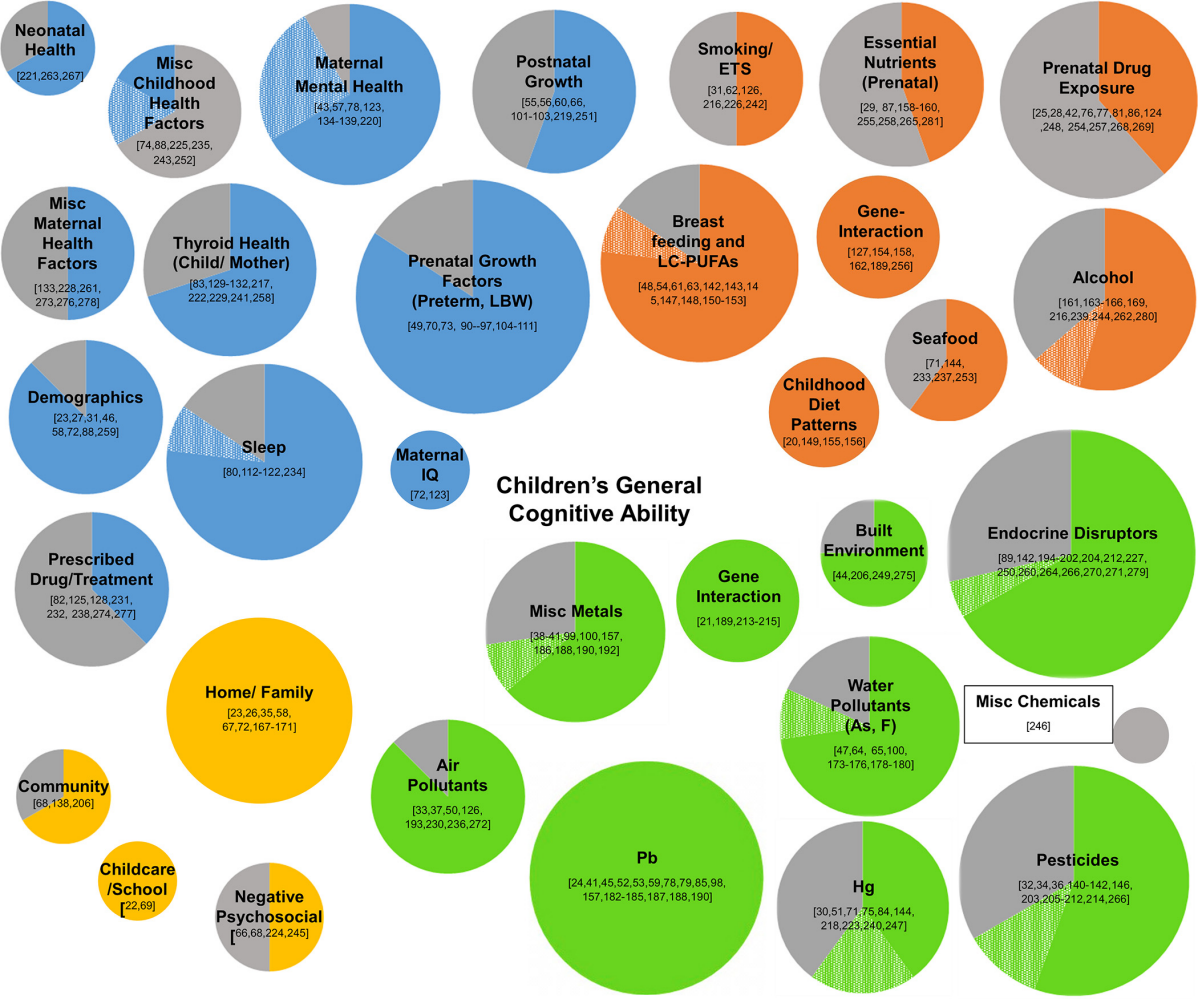
Factors Associated With General Cognitive Ability Further Divided Into Sub-categories. The color of the circles corresponds to the larger categories in which these sub-categories are grouped (inherent: blue; behavioral: orange; social environment: yellow and physical environment: green). The size of the circles is in proportion to the total number of publications included in the review (n = 258). The colored section in each circle represents the proportion of publications within each category that found a statistically significant association with general cognitive score (solid color–*P*<0.05; pattern – 0.05<*P*<0.10). This graphic is adapted from Strina et al. [282].

This review summarizes the myriad factors that can influence general cognitive outcomes during childhood. In summary, 150 studies investigated 110 possible stressors of general cognitive ability. This number does not include factors which were included as IPs in multivariate analyses. An overview of the potential stressors grouped into four broad domains–inherent determinants, behaviors, social environment and physical environment—shows that the body of literature included in this review is largely focused on negatively associated inherent factors of the mother or child or chemical exposures present in the child’s physical environment (Figs 2 and 3). Of the 33 individual or grouped factors, only three were investigated in a large number of studies (i.e., ≥10)—Pb, Hg and breastfeeding (Fig 3).

Factors with evidence of an association with general cognitive ability (i.e., consistent associations, ≥60% consensus) were not limited to already well-recognized factors, including cognitive stimulation and supportive behavior from parents, which impart beneficial effects, and childhood Pb exposure, low birth weight and early preterm birth, which impart negative effects. A consistent, positive effect was also seen with social support provided to the mothers during pregnancy, the type and quality of child care, maternal occupational class, maternal intelligence, and duration and exclusivity of breastfeeding. Evidence of a negative association also included potential indoor NO_2_ exposure, prenatal PAH exposure, postnatal fluoride or Mn exposure, belonging to a minority racial/ethnic group, multiple gestation, pre-pregnancy BMI, prenatal stress, and having siblings. It is important to note that these conclusions are only indicative of the degree of consensus of the direction of association, and not on the strength of association, which would require a meta-analysis for each stressor.

These results suggest that the positive impacts on measured cognitive ability are largely attributed to non-chemical stressors related to the mother and child’s social environment and personal decisions and inherent factors of the mother. On the other hand, declines in cognitive score are attributed to both chemical and non-chemical stressors within a child’s total environment. This review suggests that it may be a combination of stressors over a child’s lifecourse that can impact his/her cognitive development or explain the true etiology in disparities in cognitive outcomes defined by race or income.

There is general agreement between agencies, organizations and academia that there is a need to examine the cumulative impacts of exposure to both chemical and non-chemical stressors over a lifecourse to better understand variations in health outcomes and disparities [283–285]. However, the translation of this theory into research, as in the case of this review, has largely had an interdisciplinary approach, where multiple stressors are being evaluated but within their respective domains–social or physical environment, chemical or non-chemical. Recent studies indicate that non-chemical stressors can interact with or modify vulnerability to neurotoxicants; thus affecting healthy brain development [286–291]. There are ongoing prospective studies included in this review that lend support to this type of research approach focused on children’s cognitive ability. However, the non-chemical moderators of interest in this review were largely inherent or behavioral determinants, with the exception of three studies which looked at social and chemical stressors together [31, 78, 206]. Additionally, the broader definition of cumulative and environmental exposures (e.g., inclusion of community level psychosocial stressors and a range of chemical stressors) and their combined impact on children’s cognitive ability need to be considered in understanding children’s cognitive health.

An overview of the most common covariates used in this study (Table 3) reveals that current research is largely accounting for individual determinants and behaviors (e.g., demographics, maternal intelligence, birth outcomes, prenatal smoking or alcohol, and breastfeeding) in ten percent or more of the studies included in this review. The inclusion of inherent factors and behaviors, such as parent intelligence and education, are not only crucial to controlling for their influence on the level of exposure, but also serve as a safeguard for researchers to avoid over-shadowing of inheritable vulnerabilities to environmental stressors. Typically, environmental stressors included as covariates were described as confounders, rather than co-exposures, and used to focus on the variations in the effect of the exposure of interest, rather than the variation in outcome. The focus on the effect of a single stressor can stray attention away from the unknowns such as additivity, antagonism and synergism of a mixture of stressors and predispositions. Additionally, the lack of use of negative social stressors or other chemical stressors as modifying factors/covariates in multivariate regression may garner decisions relevant to blanket policies/interventions that may not be sufficient to protect children additionally facing a socially/economically disadvantaged environment which may make them more vulnerable to the stressor of interest.

**Table 3:** Covariates Used Across Publications Included in the Review.

Rank	Covariate	Frequency of Use (%)
1	Gender	56.2
2	maternal education	51.2
3	maternal intelligence	31.8
4	social/occupational class	29.8
5	maternal age at birth	28.3
6	cognitive stimulation	25.6
7	exact age at cognitive assessment	23.6
8	parity/birth order	21.7
9	birthweight, prenatal smoking	21.3
10	gestational age	20.2
11	breastfeeding	19.4
12	race/ ethnicity	15.9
13	marital/relationship status	15.5
14	prenatal alcohol	13.2
15	psychologist/evaluator	12.8
16	geographical location	9.7
17	paternal education, parent anthropometry	9.3
18	prenatal environmental tobacco smoke (ETS)	8.9
19	Income	8.5
20	child's physical health, postnatal ETS	6.6
21	birth length, parent employment, child's diet	5.4
22	maternal physical health	5.0
23	siblings, fetal/infant health, preterm/low birth weight, postnatal lead, language, resources/adverse living	4.7

### Limitations of the Study

The main limitation of this study is the considerable heterogeneity across studies due to different methodological approaches (i.e., timing of exposure and outcome, variety of cognitive assessment tools, statistical methods) that were allowed in the inclusion criteria. This limitation allowed for inconsistent results in a large number of determinants explored (Table 2), as well as difficulty in identifying critical windows of exposure for any specific stressor. The diversity in statistical methodologies alone (e.g., measurement units, data transformation) proved to be a limiting factor in making qualitative comparisons across studies for a single factor in this review; possibly hindering the ability to derive summary effects for each factor and further comparisons of effect size between factors using more formal statistical analyses.

A second limitation in this study was that only published data was used, subjecting the results to publication bias. Any effort to identify the real world effect of any stressor, with consideration of the total environment, is limited by the available data and the assumptions made by each researcher. In general, little or no data are ever published when investigations of possible stressors produce non-significant associations; thereby limiting new research from fully understanding potential moderating effects of stressors and possibly inflating summary results for each stressor in this review. This bias may have also made it challenging to identify or rule out factors that were sparsely considered. However, the approach used in this study provides the best overview of evidence that is available to describe the current state-of-the-science.

Another limitation in this study was the small number of social stressors identified in this review as compared to inherent factors, behaviors and chemical stressors which may have resulted from omission of searchable databases that may have been a better source of psychological or child development publications than PSYCInfo. The cutoff point for inclusion of studies published within the last ten years and exclusion of non-observational studies may have also limited the number of social stressors associated with this specific endpoint.

Lastly, the use of general cognitive scores as the end point in this review may not allow for identification of every single stressor or even the most important stressor because it is an average of cognitive performance over multiple domains. Certain factors that target a specific domain as opposed to having a blanketed effect over all the domains, which may help to explain inconsistencies and null effects across studies and may overlook stressors that selectively impair a specific domain. Therefore, it is important to note that the relative importance of a stressor in this study may differ for other endpoints. However, this endpoint was the one most consistently reported across studies and allowed for a greater number of factors and studies to be examined in a comparative analysis.

### Challenges and Future Direction

A major challenge in children’s research, and particularly neurodevelopment, is defining vulnerability and susceptibility so that the research community may move forward effectively in addressing the challenge of children’s exposures to chemical and non-chemical stressors and the impact on health and well-being. Childhood is defined as a sequence of unique stages described by distinct anatomical, physiological and behavioral characteristics that create variations in vulnerabilities to the environment [292]. During childhood, the brain itself is resilient and malleable, making it difficult to pinpoint an effect from a single exposure, as this may not determine the final outcome. This literature review suggests that the difficulty in identifying clear determinants of cognitive development is likely a result of the complexity with defining a child’s total environmental exposures.

This review highlights the current body of research from exposure and social sciences that have identified several factors associated with cognitive ability, with the exception of non-chemical characteristics of neighborhoods (e.g., noise, crime, social capital) and physical features of the built and natural environments (e.g., green space, food deserts, design and integrity of homes, schools and neighborhoods). Only three studies evaluated non-chemical stressor characteristics at the neighborhood level, non-chemical stressors or of the broader physical environment [44, 68, 206]. The limited research in this area may be due to the lack of quantifiable and consistent measures for neighborhood level determinants that have not been normally explored in exposure science. Additionally, non-chemical features of the physical environment are commonly considered a source of either stressors or protective factors for child development with differences in exposure being linked to social aspects of the environment (i.e., income, social class) [13, 293], rather than a determinant of health itself. A better understanding of the direct and indirect influences of the broader context of children’s environments that includes both school and neighborhood settings, as well as the built and natural environments, may be important for understanding cognitive development and associated disparities [14, 294].

## Conclusions

Cognitive development is a dynamic process, constantly changing in response to interactions with the total environment. This scoping review identified several determinants of general cognitive ability including inherent factors, behaviors, chemical stressors and family-related social stressors. Areas with limited data included distal sources of psychosocial stressors beyond those within the family and non-chemical stressors in the natural and built environment. Within this review, researchers have tackled looking at cumulative exposure by looking at multiple chemical stressors within a defined group and the interactive effects between individual determinants and chemical stressors. Few studies examined psychosocial and chemical stressors together. Given the complexity of cognitive development, the pathway(s) leading to this outcome may be as difficult to understand, considering the ubiquitous stressors children are faced with, especially those in disparate environments. A holistic approach that considers the interplay between chemical and non-chemical stressors in the built, natural, and social environments over a lifecourse can help to elucidate the true effects of key stressors that shape cognitive development.

## Supporting Information

S1 PRISMA Checklist PRISMA Checklist. (DOC)

S1 Table Summary of Publications that Met Review Criteria. Publications are grouped by study and country.
(XLSX)

S2 Table Factors Examined in Only One Study and Their Reported Association with General Cognitive Ability. (XLSX)
